# Spirit of the Foreign Periodicals

**Published:** 1841-04-01

**Authors:** 


### Spirit of the Foreign Periodicals.

### On the Alterations of the Blood in different Diseases.

In the last number of the *Medico-Chirurgical Review*, page 196, will be found a lengthened abstract of the important Memoir of MM. *Andral* and *Gannaret*, on the changes of the blood—more especially as regard the relative quantities of its fibrine, globules, and serum—in different classes of disease. We then pointed out the importance of such enquiries, mentioned some of the useful inferences that may be deduced from the results already obtained, and suggested that, in all probability, the most valuable discoveries in practical medicine will be made by following out a rational system of Humoral Pathology.

We are glad to find that some of the ablest men in France are beginning to entertain similar views to those which we have uniformly advocated in the pages of this Journal, and are giving their powerful aid in disseminating more just views respecting the necessity of attending to the conditions of the fluids in various classes of diseases. The names and high authority of *Andral* and of *Rayer* cannot fail to carry great weight with medical men of every country, and more especially with their own compatriots.*

In a recent number of the French Journal *Esculape*, we find a memoir by a M. *Monneret*, entitled " Remarks on the Alterations of the Blood," of which we propose to give an analysis. He commences with an allusion to the decay of the Broussaian doctrine.

" For some time past there has been a decided re-action against the doctrine of *Broussais*, and the humoral medicine of the ancient physicians is no longer treated with that contempt and air of superiority which have characterised many modern pathologists.

We have discovered, though rather late, that we have been following in an erroneous path, by adopting with too much confidence the medical legislation of the Val-de-Grace ; and at length many have been and are retracing their steps. We are not, however, about to return to all the vagaries of the old school, but will only assent to established truths, which actual observation may verify at any time.

Such is the direction which medical enquiries are taking at the present time.

Instead of limiting our attention to the lesions of the solids exclusively, without tracing them up to the physiological cause on which they depend, medical men are now engaged in studying the alterations of the blood and the other fluids of the body, and following out a method of enquiry which cannot fail of leading to very important results.

The recent memoir of MM. *Andral* and *Gannaret* has shewn what may be done, and has already contributed to throw considerable light on the pathology of many diseases."

M. *Monneret* very justly remarks that, until within the last few years, medical men have occupied themselves too much with the minute chemical analysis of the blood, and have too often overlooked, in consequence, the more obvious and more important changes in the relative proportions of its principal constituents. However useful the elaborate researches of *Lecanu* and other distin-

* We may refer our readers to some remarks on the *humoral* pathology, so to speak, of rheumatic and gouty affections, in the last number of this Review ; vide art. *Bouillaud* on Articular Rheumatism.—*(Rev.)*

guished chemists may be, we must remember that, for practical purposes at least, the examination of all the fluids of a living body should be conducted in a physiological rather than in a merely chemical manner.

Before alluding to the changes which the blood exhibits in different diseases, let us dwell for a moment on the normal constitution of this vital fluid.

When blood is allowed to rest, it separates into two parts, the clot and the serum—the former consisting of the red globules and the fibrine, and the latter of water, which holds in solution albumen and various saline matters.

The fibrine of the coagulum forms a meshwork, in the interstices of which are entangled or enclosed the numerous globules to which the blood owes its colour, and also a quantity of serosity similar to that in which it, the coagulum, floats. The principal salts which along with albumen are dissolved in the serum, or watery portion of the blood, are the muriates of potassa and soda, the sulphate of potassa, the carbonate of soda, and the phosphates of soda and lime. The globules seem to consist of albumen united to a colouring matter, to which the name of *hæmatosine* has been given.

The relative proportion of these different constituents in healthy blood may be stated thus :—

| | | | |
|---|---|---|---|
| 1. Fibrine .. .. .. .. .. | 3 | parts | The Clot. |
| 2. Hæmatosine .. .. .. .. | 2 | ,, | |
| 3. Solid albumen of the globules .. .. | 125 | ,, | |
| 4. Liquid and dissolved albumen .. .. | 68 | ,, | The Serum. |
| 5. Saline matters .. .. .. .. | 12 | ,, | |
| 6. Water .. .. .. .. .. | 790 | ,, | |
| | 1,000 | parts. | |

The relative proportions of these constituents of the blood are found to vary much in different classes of diseases; and these variations are so uniform and constant, that we are completely warranted in asserting that there is a strict relation between them and the nature of the morbid process that is going on in the system. For example, that a most characteristic feature of all the genuine phlegmasiæ is a superabundance of the fibrine of the coagulum, has been long known, and is most clearly proved by the researches of MM. *Andral* and *Gannaret*.

It appears also, from these researches, that in the genuine fevers, such as typhus, the various exanthemata, and certain vague or ill-marked pyrexiæ, the quantity of the fibrine is not increased, but is either stationary or somewhat below the normal standard. Again, chlorosis is an example where the proportion of the red globules is sensibly diminished; and in the *morbus Brightii* the chief morbid change seems to consist in a diminution of the quantity of the albumen held in solution by the serum.

These facts have indeed been long known to most practical men, who have not allowed their minds to be fettered by the exclusive doctrines of the solidists; yet still much credit is due to MM. *Andral* and *Gannaret* for the statistical illustrations which they have adduced in support of them. One, however, of the most unexpected of all the results of their labours has been to shew that in *phthisis,* at least in certain stages of it, there is almost always an excess in the proportion of the fibrine of the blood. M. *Monneret* says on this subject :—

" When the tubercles are still crude, the increase of the fibrine is scarcely appreciable; when they begin to soften, it is more marked; and at length, when vomicæ are formed, the proportion of this element sometimes rises to six parts in the thousand.

The red globules, on the other hand, follow the very opposite direction; their period of decrease is progressive from the commencement to the close of the disease: the difference often exceeds twenty parts. The disease is therefore represented in its complexion by the increase of the fibrine on the one hand, and by the diminution of the albuminous globules on the other. To the one of these alterations corresponds the complication, and to the other the pathological condition of phthisis."

Such is a very important discovery of modern humoral pathology—provided it be confirmed by the researches of others. However this may be in reference to the state of the blood in phthisis, the statements of MM. *Andral* and *Gannaret* are, we believe, entitled to implicit confidence on the important question of the pathology of fevers.

We cannot have a more convincing proof of the error of *Broussais* and his disciples, in regarding typhus and other fevers as of the nature of the genuine phlegmasiæ, than what is afforded by the researches of our authors. For while in the latter classes of diseases, the proportion of the fibrine of the blood is invariably increased, in the former it is never so, and in not a few cases is even sensibly diminished; whereas the proportion of the red globules is at the same time either unaffected, or somewhat higher than it is in health.

The following is a summary of the results obtained by MM. *Andral* and *Gannaret's* examination of the blood in different forms of fevers.

In *simple continued fevers,* no increase in the quantity of the fibrine is observed either during the precursory stage, or when the disease is fairly formed: in several cases it is sensibly diminished. On the contrary, the proportion of the red globules is almost always increased; this increase being sometimes very considerable.

In *typhoid fevers* also, although there is a decided inflammatory complication of the gastric and intestinal mucous membrane and glands, we never observe any increase in the proportion of the fibrine at any stage of their existence—a demonstrative proof, if others were wanting, that these fevers cannot be justly regarded as phlegmasiæ, and therefore that the terms *gastro-enterite, mesentero-enterite,* &c. are most fallacious and improper.

(We wonder how our friend M. *Bouillaud* will reconcile these facts with his practice of bleeding *coup sur coup*: doubtless, he is too adroit a debater not to be ready with an answer; for never did the poet's line

—— e'en though vanquished, he could argue still, apply to any one more truly than to the physician of La Charité.—*Rev.*)

In *eruptive fevers,* the genuine exanthemata, the proportion of the fibrine in the blood is almost invariably below the normal standard. That of the red globules is occasionally, as in many cases of scarlatina and rubeola, considerably increased. It appears therefore that the existence of a cutaneous phlegmasia is not in itself sufficient to induce an inflammatory condition of the blood, or that its influence is counteracted and neutralised, so to speak, by a specific principle on which the exanthemata depend.

In *intermittent fevers,* the blood does not seem to undergo any appreciable alteration. Does not this prove that this class of diseases results from a general disturbance (ebranlement) of the organism, and that the nervous system acts an important part in their production?

Another important result of MM. *Andral* and *Gannaret's* researches is, that, in cerebral congestions and hæmorrhages, *the condition of the blood is very nearly the same as it is in simple fevers.*

In not one of fifteen cases of apoplexy, examined by these gentlemen, was the proportion of the fibrine at all increased, and in some of them it was diminished; whereas that of the red globules was found to be above the normal standard in every instance without exception........ "This double alteration, which is especially remarkable at the commencement of the disease, is characteristic. It proves that the less fibrine there is in the blood, the less coherent it becomes, and therefore that cerebral hæmorrhage is dependent rather upon an essential modification in the elements of the circulating fluid, than upon any lesion of the solids.

Still there is nothing absolute in this conclusion; for in numerous cases it happens that certain pathological conditions of the solid parts induce apoplexy, and then the blood does not exhibit the abnormal conditions which we have mentioned."

So much for the humoral characters of inflammatory, febrile, and some congestive and hæmorrhagic diseases. We come now to a very opposite class of maladies—those in which there is a striking diminution in the proportion of the red globules contained in the blood. In chlorosis, especially, and indeed in almost all cachectic states of the system, by whatever cause induced, this is remarkably the case.

In a case of diabetes the proportion was found to have fallen from 127, the standard of health, to 86; in a case of dropsy, connected with disease of the heart, to 68; and in various cases of chlorosis to 77, 70, 60, 50, 46, and in one instance to 38. In no other disease has the relative quantity of the red globules been found so low as in chlorosis. The gradual increase in this quantity during the administration of ferruginous preparations was repeatedly ascertained. How is this effect of steel to be explained? We must confess that we are unable to say: unless, indeed, by attributing it to the improvement of the general system, which is then enabled to bring back the process of sanguification to a healthy state.

With respect to the last division of diseases according to the humoral nosology—those in which the characteristic feature is an alteration in the proportion of the albumen dissolved in the serum—we have no new facts to mention. It is chiefly in *Bright's* disease of the kidneys that this change is most remarkable; for it would seem that the larger the quantity of albumen that exists in the urine, the less will be found in the serum of the blood. As yet, no experiments have been made as to the state of the serum in other diseases, in which this morbid condition of the urine is known to exist.—*L'Esculape.*

---

### M. Beau on Auscultation.

In our last number we gave an abstract of M. *Beau's* first memoir, and directed our readers' special attention to the novel and very ingenious views which he has propounded. It will be remembered that his leading position is, that the respiratory murmur of health, as well as the various modifications which it presents in a state of disease, are owing to the retrograde resonance of what he calls the *glottic sound* in the bronchi and cells of the lungs, and not (as *Laennec* and all other auscultators have supposed) to the friction of the air on the parietes of these tubes and cells. According to this view, the glottis, and not the lung, is the proper seat of the vesicular murmur.

He draws what he considers a powerful argument in favour of his opinions, from the admitted fact that, in certain cases, this vesicular murmur is entirely inaudible, although the respiration continues to be regularly performed.

........ "This difficulty had occurred to the mind of *Laennec* himself, in reference to the *glottic* crowing or whistling sound of hooping-cough. The following passage from his great work may be here quoted:—'The whistling and prolonged inspiration, which constitutes the pathognomic character of this disease, appears to take place altogether in the larynx and trachea. We cannot hear either the sound of the pulmonary respiration, nor the bronchial respiratory sound, even in those parts of the lungs which, a few moments before, and after the hoop or kink, gave out a distinct puerile murmur. This *phenomenon*

can only be explained in one of two ways:—*either* by supposing that there is a momentary sanguineous or serous congestion and consequent swelling of the mucous membrane of the bronchi, sufficient to obstruct these canals; *or*, that there is a spasmodic contraction of the bronchi, which would produce the same effect." P. 188, ed. 1826.

Now *Laennec* would never have hazarded such a conjecture, had he observed this *phenomenon* (as he calls it) in a case of organic, and not merely in one of spasmodic, contraction of the larynx. What, however, had escaped his notice, has been distinctly alluded to by Dr. *Stokes* of Dublin, and by M. *Barth.* The former of these gentlemen says, in his Treatise on the Diseases of the Chest;—"In the diseases of the larynx, the vesicular murmur becomes feeble in proportion to the degree of the obstruction. It is observed in some cases to be weak, or entirely absent, over the whole chest."

The latter, (vide Archives de Medecine, p. 227, 1838,) thus expresses himself: —"The vesicular respiratory murmur may be diminished, or altogether abolished, on both sides of the chest by any lesion which is capable of contracting the calibre of the upper part of the air passages."

Neither of these propositions is strictly correct. For, in truth, we frequently meet with cases of contraction of the laryngo-tracheal tube, in which the respiratory murmur is not only audible, but even louder than in health: and the reason of this is, that the passage of the air being somewhat impeded at the obstructed point gives rise to an exaggerated blowing sound. It is only when the disproportion between the volume of air and the laryngo-tracheal obstruction is more considerable, that this sound loses its normal blowing character, and acquires a snoring or whistling tone. Under such circumstances the pulmonary resonance undergoes the same change; and hence the usual respiratory murmur is no longer audible.

This exaggeration on the one hand, and absence on the other, of the respiratory murmur in the lungs are sometimes observed, alternating with each other, in the same person. In a case of syphilitic swelling of the larynx, under the care of Professor *Fouquier*, the laryngeal sound was observed to vary according to the rapidity of inspiration; for it had a blowing character, when the air passed slowly through the contracted point, but immediately acquired a metallic-like snoring character, when the breathing was quickened. On ausculting the chest, these two different sounds could be distinctly observed to alternate with each other.

The same thing was noticed in the case of a man, who, having undergone the operation of tracheotomy for a contraction of the larynx, was obliged to breathe through a canula. In him there was heard a vesicular murmur, only in an exaggerated degree, because the orifice of the canula being rather small gave rise to a strong blowing sound; but when the canula was obstructed, and he was obliged to breathe by the mouth, a loud snoring sound was produced at the contracted point, and this snoring sound was reverberated backwards through the entire chest, overpowering and taking the place of the normal vesicular murmur.

These alternations of absence and of exaggeration of the normal respiratory murmur are frequently observed in hysterical patients affected with spasm of the glottis.

According as the glottis happens to be more or less contracted, there is a whistling or only an exaggerated blowing sound audible; and it will then be found that the lungs give out on auscultation at one time a whistling or sibilant sound which completely hides the ordinary vesicular murmur, and at another time an exaggerated blowing sound, to which the epithet *puerile* has been given by most writers.

To explain these phenomena according to the theory of *Laennec*, we must suppose that in the cases of considerable contraction of the larynx, where the vesicular murmur is replaced by an abnormal sound, the air does not penetrate into the cells of the lungs; and on the contrary that in cases of less considerable contraction, when the blowing sound is merely exaggerated, or, in other words, has become puerile, the air penetrates in a superabundant quantity: —an explanation which cannot surely be maintained by any unprejudiced reasoner.

M. *Beau*, after some further remarks on this part of his subject, proceeds to examine the subject of the various *rales* which have been described by auscultators. These sounds, he impresses on his readers, are very different from the abnormal modifications of the respiratory murmur, and of the voice, to which we have hitherto alluded; for they are solely and altogether the results of morbid changes in the bronchial tubes and pulmonary cells themselves, and not of the retrograde resonance of any glottic sounds. He divides them into two classes—the *vibratory*, or *dry*; viz. those which consist in a more or less prolonged vibration of the air in consequence of an obstruction in some point of the air passages, and the *bullar*, or *moist*, viz. those arising from the rupture of bullæ, of larger or smaller dimensions in different cases, produced by the air traversing a fluid obstacle in these tubes.

The *Vibratory Rales.*—The most remarkable of these are well known as the sibilant, the sonorous or grave, and the snoring *rales* of *Laennec* and other authors.

........ "The existence of these different kinds of vibratory rale is in general very transitory. A single expectoration or act of coughing is often sufficient either to cause them to cease altogether, or to change them into a form different from that which was present at first: thus the sibilant *rale* becomes sonorous, snoring, or blowing; or perhaps one of these last *rales* acquires the sibilant character, soon to be again transformed."........ "The various vibratory rales may be of the same duration as the corresponding respiratory movements, or they may be shorter than these. The same rale may be heard both during inspiration and during expiration; or it may be present during one act and absent during the other. In the latter case, it is during the expiratory movement that the rale is most frequently heard—hence the number or variety of the expiratory is greater than that of the inspiratory rales.

To explain these last facts, we are obliged to admit that the obstacle in the bronchi, which sets the air in vibration, does not necessarily exist during both acts of respiration; and, as we have just stated, that the expiratory rales are in general more frequent than the inspiratory, it follows that the air meets with more impediments in its escape from, than in its entrance into, the bronchi.

The vibratory *rales* may have their seat in every point of the bronchial tree; but the different forms of these *rales* usually affect tubes of a different diameter. Thus the *sibilant rale,* conveying the sensation of an attenuated stream of air, is most frequently heard in the small bronchi; the grave or *sonorous rale* in bronchi of a middling size; and the *snoring* or loud blowing rale in the larger bronchi."

............ M. *Beau* attributes the origin of the vibratory *rales* in almost all cases to an obstruction of some of the bronchial tubes from the presence of viscid mucus, and very rarely to any actual swelling of their lining membrane. His chief reason for this opinion is, that "these rales seldom continue for any length of time the same, but are continually changing from one tone to another, from the sibilant to the sonorous or the snoring, and back again from the latter to the former. Now we get rid of this difficulty, if we suppose that the operating cause is the presence of viscid mucus in the air-passages; for, as this mucus may be displaced by any effort of the breathing, the character of the existing *rale* will depend chiefly upon the size of the tube, or tubes, which may be partially obstructed. In what other manner can we explain the occurrence of *rales,* which are audible during one only of the acts of breathing, and not during the other? The intermittent obstacle, which gives rise to them, cannot well be supposed to be a swelling of the lining membrane of the bronchi; on the contrary, how easily explicable is the phenomenon on the supposition that some of these air-tubes are partially obstructed by a portion of mucus, which having the form of a *languette,* and its edge being raised sometimes towards the larynx, and at other times towards the pulmonary cells, may be most aptly compared to a half-opened valve, which throws the air into vibrations either during inspiration or during expiration."

M. *Beau* accounts for the greater frequency of the expiratory than of the inspiratory rales on the ground that the air must encounter a greater degree of obstruction in its exit from, than in its entrance into, the small bronchi and the pulmonary cells, from the circumstance of the whole tissue of the lungs being more or less contracted during the act of expiration by the descent of the ribs and the rising of the diaphrgm.

In consequence of this compression, which necessarily affects both the blood-vessels and the air-tubes of the lungs, the interval between the loose edge of the mucous obstacle and the corresponding wall of the tube must be more or less diminished, and the intensity of the *rale,* produced by the passage of the air, will therefore be proportionately increased.

As to the cause of the crepitant *rale*—the pathognomonic auscultatory sign of pneumonia—M. *Beau* suggests a novel interpretation :—

...... " It is generally supposed that this sound is caused by the rupture of extremely small bubbles of mucus in the pulmonary vesicles. But if we consider that the sound is not at all modified after a fit of coughing, and also that it is distinctly perceptible in many cases of pneumonia before any expectoration takes place, it may be fairly asked whether it may not rather depend *on the friction of the pulmonary vesicles,* which, like the pleura, pericardium, and the synovial membranes, are probably somewhat *dried* (dessechées) by the existing inflammatory action."

(The cases are certainly not parallel; these tissues being serous, whereas the surface, at least of the vesicles, is of a mucous character.—*Rev.*)

" Besides, a sound, exactly similar to the crepitant *rale* of pneumonia, is readily imitated by blowing air into the lungs of a sheep, which have lost part of their moisture. I should not hesitate, adds, M. *Beau,* to adopt this explanation of the crepitant *rale,* if it was once proved to me that inflammation has the effect of drying the pulmonary vesicles."........

After commenting at considerable length on the various other *rales,* M. *Beau* proceeds to treat of the auscultatory phenomena which are accompanied with a *metallic sonorousness* :—He says,

" *Laennec* was the first who discovered that when either a large cavern in the lungs, or the cavity of either pleura, contains both gaseous and liquid contents, we not unfrequently hear sounds which are altogether similar to those which a glass or metal vessel gives out when struck.

We cease to wonder at such an occurrence, when we find that it is not peculiar to the chest; metallic sounds being often heard in the cavity of the stomach, not only when the ear is directly applied to the abdomen, but when we listen at a considerable distance from it. We must confess that we do not sufficiently understand the exact conditions which are necessary for the production of this phenomenon, even in the case of inorganic vessels, whether of wood or of metal or glass. This, however, we know, that unless the air within them is thrown into vibration, they emit no sound; and so it is with abnormal cavities within the thorax which gives out any metallic resonance :—it is never heard, unless a vibration takes place in their interior.

Now such a vibration may arise from several causes :—thus, 1. It is produced when a patient, in whom such an abnormal cavity exists, is briskly shaken by the shoulders, and the liquid contents of the cavity are made to strike against its parietes: the sound produced by this succussion is sometimes very loud and may be heard at a considerable distance; it is *the metallic sound of succussion.*—2. The voice, the act of coughing, &c. may occasion in the cavity a resonance quite similar to that heard by speaking in a well or in a large deep vessel; this is *the metallic echo.*—3. The resonance of the glottic souffle may give rise, by the vibration of the inspired air, to a sound similar to that produced by blowing into a large empty vessel; this is *the amphoro-metallic sound.*—4. Lastly, the mere rupture of large bullæ of air in the abnormal cavity may produce a sufficient vibration to cause a short and irregular metallic sound; this is *the bullar tinkling.*

It is therefore apparent that metallic sonorousness is not so much a special abnormal sound, as a peculiar tone (timbre) which the normal or abnormal respiratory sounds acquire under certain circumstances. Thus the *metallic echo,* and the *amphoro-metallic* sound, are only peculiar resonances of the voice and of the glottic *souffle*; while the *bullar tinkling* is nothing but a *rale* produced by the rupture of large air-vesicles or bullæ, acquiring a metallic resonance in consequence of the property of the cavity wherein it takes place."*—*Archives Gen. de Medecine, Août.*

---

### On the Electrical Actions in Living Bodies.

Dr. *Riche,* a physician at Obernay, is the author of the following remarks, on this curious and most interesting department of physiological science. He commences his paper with laying down these positions:—

1. "Every molecular action, whatever be its cause, produces a state of electrical tension.

2. Every time that the electrical tension is destroyed by neutralization, the recomposition of electrical states, &c. there is a production of light (phosphorescence) with or without appreciable heat; and *vice versâ.*

3. In bodies which are good conductors, the neutralisation is instantaneous and consequently invariable; whereas, in those which are bad conductors, the neutralisation takes place more slowly, and becomes sensible to our senses and our instruments.

4. The electrical tension of a body is the sum of the electrical tensions of its molecules.

5. Every time that an electrical current encounters an obstacle in its transmission through any part of the body, there is a production of heat. A calorific current produces, in the same circumstances, an electrical current. Light developes both the one and the other in different degrees.

6. An alternating series of molecules, which are more or less perfect conductors of heat and electricity, produces, by their mere position, an electrical or calorific current.

7. Lastly, electricity, caloric, and light, seem to be only different modes of manifestation of molecular movements."

---

* It is physically possible that a drop may fall from the top of the cavity upon the fluid below, at the moment when the patient lifts himself up from the horizontal position, and that its falling may give rise to a metallic sound; but then it is difficult to conceive that a sufficient number of drops should be falling, one successively after the other, to enable us to explain the circumstance of this auscultatory phenomenon being heard for a considerable length of time.

Dr. *Riche* proceeds to develope with great ingenuity his views of the manifold operations of living bodies, such as the functions of muscular contraction, of animal heat, of secretion, &c. all of which he regards as so many manifestations and results of electrical agency. In short, he regards the whole living organism as a wonderful electrical machine, obeying the general laws which pervade the universe, but governed and controuled in all its changes by a higher immaterial principle, the nature of which is beyond our ken.

........ "Keeping in view," says he, "the above preliminary positions, we may well ask, what bodies in nature unite in themselves more of the conditions necessary to produce the electrical tension and neutralisation than those which are living and organised?

A vesicular, cellular, and vascular organisation; fluids charged with saline, acid or alkaline principles, and holding in combination phosphorus, sulphur and certain metals; an incessant composition and decomposition from the first moments of life to the period of death; a circulation of fluids through vessels of every nature and in various directions; a consumption of a large quantity of oxygen, the most favourable condition for the development of electrical action; lastly, a nervous system diffused or centralised, isolated, transmitting with the rapidity of lightning impressions from without inwards, and reactions from within outwards. Is not every thing combined in such a system for developing a maximum of molecular action?

The torpedo, and other species of electrical fish, display the effects of this action most vividly in consequence of the peculiar apparatus with which they are provided. If it be said that the peculiarity of these organs proves that they are an exception in the animal kingdom, it may be justly answered, that these organs are merely electric condensers; that the source of their electricity is not in them, but that this is transmitted by the large and numerous nerves of the brain; for we know that any mechanical irritation applied to the brain or its nerves augments the intensity and frequency of the shocks, and that the division of these nerves suspends their power altogether. Do we require fresh proofs?

By experiments on frogs, it has been clearly shewn that there are in these animals electrical currents, which are independent of all external and foreign excitement or stimulus.

These currents produce muscular contractions, when they proceed from the nervous trunks to their ramifications, and sensations when they proceed in the opposite direction from the ramifications to the trunks.

Again, cats, horses, dogs, and man himself, often exhibit visible signs of electricity from exposure to the sun, or in consequence of friction of the surface or after muscular exertion: even electrical shocks, attended with sparks, have been occasionally given out by certain persons.

An electrometer exhibits signs of electricity, when it is brought in relation with a person placed on an *isoloir*. The mere approach of the finger will sometimes attract a needle which is finely suspended.

Lastly, in the operation of acupuncture, signs of an electrical condition have been distinctly perceived.

Now, what are the conditions which produce such sensible electrical effects in living bodies?

The blood is formed of vesicular globules, inclosing a dense coloured liquid, and floating in a liquid which is less dense and which combines all the conditions of electrical conductibility.

Through the vesicular parietes of these globules, there is incessantly going on an alternate endosmosis and exosmosis;—actions which are always accompanied, it is known, with electrical currents: the blood, therefore, is electrical by its very constitution.

Moreover the blood, being subjected to a continual circulation, must exercise a certain amount of friction on the walls of the vessels which contain it—a new source of electrical tension, which is then transmitted to the nervous twigs distributed on their outer coats.

Again, the blood traverses the lungs, gets rid of various excrementitious products, and is electrified anew by this very process of elimination: it receives the influence of the external air with all its conditions of vitality—that is to say, of the oxygenated luminous air, possessing an electrical tension which is variable according to the bodies dissolved or suspended in it. The heat developed by respiration, circulation, and muscular contraction, tends to increase the electrical tension of the blood, by the movements thereby incessantly communicated to all its molecules.

This heat itself has its origin in the innumerable electrical discharges which are taking place every where and at every moment, and in the obstacles which the electrical currents meet with in passing from one molecule to another, from one liquid to another, and through the numerous cellules, membranes, and the parietes of vascular tubes.

To these various sources of electrical generation we may add the functions of digestion, of secretion, and excretion: and even then we shall have but a most imperfect idea of the constant decomposition and recomposition which are going on at all points of the living frame."

........ "Will it be permitted us to regard the ganglionic nerves, arranged in inextricable meshes around the arterial trunks and ramifications, as conductors which transmit to the nervous centres the electricity developed in the parenchymatous structure of the viscera? According to our views, this electricity, accumulated and concentrated in the nervous centres, is destined to the production of muscular contraction, as well as to the maintenance of a uniform animal temperature, and to the regulation of the acts of organic composition and decomposition.

We admit, with Professor *Duges* of Montpelier, that muscular contraction is the result of a series of electrical discharges, which rapidly succeed each other on the contracted muscles. The heat produced by exercise, and the fatigue which follows upon it, seem to confirm this view of the matter; for the heat is the result of the neutralisation of electricity, and the fatigue is the result of nervous exhaustion."

Dr. *Riche* does not wish his readers to suppose that he regards nervous and electrical action to be strictly identical, or that the one term can be appropriately substituted for the other. Although there are numerous relations and points of resemblance between them, the former is in some mysterious manner modified by the living principle, so that we can never hope to imitate its phenomena by any artificial contrivance, however ingenious and complicated. Having again expressed his opinion that every muscular effort is the result of repeated electrical discharges in the muscles called into play—an opinion which is strongly confirmed by the circumstance, that in electrical fish the body of the animal is always observed to be made tense and contracted before it gives a shock—Dr. *Riche* suggests an explanation of various morbid states of the nervous system on the doctrines now propounded.

......... "The derangements of innervation may be considered—1, as the result of an insufficient or of an excessive electrical tension;—2, as the effect of an impediment to the transmission of electrical currents;—3, as dependent upon a feebleness of the will or on a depraved instinct, or on both together. (A well disciplined moral education, exercising, directing, and controuling the will, giving it the mastery over the body, and over each of the organs in particular, and keeping the instinct within limits compatible with the moral and physical welfare of the individual, is the best preservative against the last-named cause of nervous diseases.)"

Our author seems to be a partial believer in Animal Magnetism. Let us hear what he says :—

"We may perhaps refer to the electricity of living bodies, whatever has been well confirmed as to the effects of animal magnetism ; for example, the sedative and exciting phenomena produced by its operation. Every time that we operate with due precaution, perseverance, and impartiality, we shall produce these effects ; there is no need of a blind credulity on the part either of the patient or of the operator. An organised body, better perhaps than any other, may be electrised by influence or by contact, through the means of electricity naturally developed in the living body, and which can be directed specially to such or such a suffering part. The experiment becomes still more easy, when the two actors are placed on insulating stools. It is the foolish pretension to produce extraordinary and marvellous effects, which some operators have put forth, that has prevented many useful results being obtained from animal magnetism."

It remains now to notic briefly Dr. *Riche's* remarks on the properties of electricity as a remedial agent.

.......... "Whenever there is an excess of electrical tension present, evidenced by tetanic contraction, by spasms and painful cramps of muscles, we should endeavour to neutralise at once this condition by an energetic shock with the Leyden jar. The same mode of employing electricity might be used with advantage in certain cases of epilepsy, catalepsy, lethargy, &c. provided always, there is no reason to apprehend any tendency to hæmorrhage or inflammation in the brain or other parts of the nervous axis.

In the treatment of palsy, it is better to give a succession of smaller shocks by induction, than by the more powerful discharge of a Leyden jar. When we wish to act deeply with feeble currents, and still more when our object is to cauterise by means of the heat produced in metallic wires by a strong current of electricity, we must have resort to acupuncture ; this too is the proper method of employing electricity for the purpose of discussing or altering the vitality of tumors and other morbid degenerations."...... *Revue Medicale.*

---

### Professor Arnold on the Functions of the Pneumo-gastric and the Accessory Nerves.

This distinguished Professor has long paid much attention to many of the disputed points in the physiology and pathology of the nervous system. The following observations on the functions of the pneumo-gastric and the accessory nerves—the 10th and 11th pairs of the cerebral nerves according to the arrangement adopted by most German anatomists—deserve our notice. They are based on the phenomena exhibited—1, by lesions of these nerves—and 2, by experiments performed on the lower animals.

The author has collected the following cases of morbid lesion from various sources.

*a.* A priest, 62 of age years, had from his youth been subject to gouty attacks. During the last seven years of his life, the stomach was the seat of his chief sufferings ; for eighteen months his principal distress was a most voracious appetite, which could scarcely be satisfied with any quantity of food. The contents of the stomach, when rejected by vomiting, did not exhibit any symptoms of digestion, even three or four hours after being swallowed. The respiration became embarrassed ; and when the dyspnœa was most severe, a whistling noise was made, as if the glottis was contracted.

The patient became much emaciated ; the pulse was natural in point of frequency, but always rather strong ; and there was never any pain felt in the chest. For several weeks before death, opium in all forms produced disagreeable effects.

The employment of galvanism generally relieved the dyspnœa. Numerous purple spots made their appearance on various parts of the body; but these vanished for some time before death.

*Dissection.*—Two pints of a deep-coloured fluid were found in each pleural cavity; the lungs themselves seemed healthy; the pneumo-gastric nerves, from the nucha downwards, were smaller and softer than usual, resembling in consistence the nerves in putrid bodies which have been subjected for some time to maceration: the left nerve was smaller that the right one.

*Swann,* who has reported this case in his work On the Local Diseases of Nerves, supposes that the fluid in the pleuræ had been effused only a short time before death. He adds that, in other two cases of death from thoracic complaints, he had found the nervi vagi considerably smaller than they are usually observed to be.

Professor *Arnold* remarks, that the symptoms in the preceding case are exactly those observed in animals after section of these nerves.

*b.* A woman, who died *poitrinaire,* had for a length of time been subject to an almost insatiable appetite.

On *Dissection,* both nervi vagi were found to be covered with minute reddish oval ganglia, of the size of small peas, and formed of the nervous substance itself, and not of the neurilema: the two sympathetic nerves were atrophied.

*c.* The next case is reported by Dr. *James Johnson,* in the *Medico-Chirurgical Review,* for July, 1836.

A lady, 76 years of age, had suffered for twenty years from pains in the head and back. When Dr. *Johnson* first saw her, she was greatly emaciated, and suffering from inexpressible anguish; she could not articulate a single word, and could scarcely swallow the smallest quantity of jelly. She experienced neither hunger nor thirst; food was introduced into the stomach by means of an œsophageal tube. She frequently complained of great tenderness in the epigastric region. Galvanism was tried, but it failed of producing any benefit. All the symptoms continued up to the period of death.

On *dissection,* the pons *Varoli,* the medulla oblongata and upper portion of the spinal cord were found in a state of *ramollissement;* the left vertebral artery, on its issue from its osseous canal, was considerably enlarged, so as to compress the corpus olivare and pyramidale of this side, which seemed smaller than their fellows on the opposite side. From the same cause the roots of the glosso-pharyngeal, the pneumo-gastric, the accessory and the hypoglossal nerves were somewhat pressed upon; their structure, however, did not exhibit any abnormal appearance. In the chest, there was discovered a small aneurismal tumor on the descending aorta, on the surface of which the left pneumo-gastric nerve was stretched, so as to take the form of the letter C. There was no contraction of the œsophagus in any part. The author, Dr. *Johnson,* was of opinion that respiration and digestion had been maintained by the intact nerves of the right side.

*d.* A case is mentioned by *Bell,* in his physiological and pathological researches on the nervous system, in which a convulsive affection of the sterno-cleido-mastoid and of the trapezius muscles was the most remarkable feature.

The patient was obliged to support his head with both hands; he complained that his body was always drawn round to one side with great force. The sterno-cleido-mastoid muscle during this time became tense as a board, and the right side also of the neck became almost equally stiff, owing to the convulsive contraction of the trapezius muscle. The head was drawn and fixed downwards, the face being turned to the left side, and the chin directed upwards. The breathing was unembarrassed, and there was no pain felt in the chest. When the spasmodic attacks were very violent, the muscles of the larynx became affected, and the patient seemed to try to expectorate something that hindered him from speaking. These attacks were brought on by drinking any fluid; it was only during sleep that they ceased entirely.

Besides these four cases, now briefly reported, we may point to other two—one published by *Cappel* (De Epilepsiâ e tumore nervo vago inherente, Helmst. 1781,) and the other by *Tilgen,* (Diss. obs. syst. fungi medullæ nervi vagi, &c. Bonnæ, 1830,—Raucedo, tussis, vomitus Ciborum incoctorum et potuum.)

Professor *Arnold* has performed numerous experiments on dogs, rabbits, chickens, and pigeons, for the purpose of ascertaining the functions of the pneumo-gastric nerves.

It is unnecessary to give the details of these, and we shall therefore condense their general results in the following remarks.

The pneumo-gastric nerve is considered by Professor *Arnold,* contrary to the opinion of many physiologists, to be a sensory nerve, and as capable of communicating impressions made on its extreme filaments to the brain; these impressions being communicated with greater or less distinctness, according to the special functions of the mucous membrane on which it is distributed.*

It communicates the sensation of hunger, and the *besoin* of respiration. It has not any *direct* influence on the secretion, or on the quantity or quality of the gastric juice, (as supposed by *Tiedemann, Brodie, W. Philip,* &c. who have alleged that the secretion of proper gastric juice is suspended by the division of the nervi vagi;) nor upon the muscular contractions of the œsophagus and stomach, nor upon the process of chymification, nor on the muscles of the glottis, nor upon respiration or the movements of the heart, nor on the heat of the animal, nor on the process of arterialisation or the conversion of the black into red blood.

But as it is a nerve of sensation, and therefore cannot continue, after being divided, to communicate to the brain the *besoin* of breathing, the respiration becomes after their section slower and slower, the arterialisation of the blood is thereby impeded, the temperature of the body falls, the blood accumulates and stagnates in the cavities of the heart, in the great vessels, and in the lungs, and ultimately the animal dies suffocated. It thus appears that the fatal effects of the section of the nervi vagi are *indirect,* and are not, as many physiologists, have supposed, attributable to any immediate paralysis of the respiratory parenchyma, or of the muscles of breathing, but rather to the *besoin* of breathing being no longer felt by the animal, and the consequent gradual retardation and ultimate suspension of this vital process.

Hence the circulation of the blood through the lungs becomes more and more embarrassed, and as the blood is but imperfectly aerated, the functions of the nervous system gradually cease. The animal dies asphyxiated; but the asphyxia or suffocation is slow and progressive, unless there be some obstruction

---

* The division of the pneumo-gastric nerve in the neck is not productive of pain; but when the superior laryngeal branch is divided, acute suffering is experienced. The more exquisite sensibility of the larynx and glottis than that of the œsophagus and stomach is accounted for by Professor *Arnold* on the ground that some of the branches of the nerve are much less connected with, or involved in, its plexuses than others. Thus the superior laryngeal branches participate little, if at all, in the *gangliform* plexus, whereas that portion of the nerve, which descends to the gullet and stomach, contributes largely to its formation.

Now it is an admitted position in physiology, that nerves, which expand themselves in plexuses, convey impressions to the brain much less quickly and distinctly than other nerves which are insulated.

of the glottis or of the windpipe—as is apt to be the case, more especially when a young animal is the subject of experiment.

The internal branch of the accessory nerve presides over the contractions of the œsophagus, and those of the glottis and stomach: it becomes united with the pneumo-gastric in the neck.

It is, therefore, very important, as well in experimenting on animals as in observing the effects of disease in man, to distinguish the symptoms induced by any lesion of these two nerves.—*Archives de Medecine, Août.*

---

### Microscopical Distinction between the Sensory and the Motory Nerves.

In a paper on the functions of the ganglionary nerves by Dr. *Remak*, in the June number of *Ammon's* Monatschrift, we observe the following paragraphs:—

"Every nervous bundle is composed of an assemblage of primary filaments, which do not communicate together, but are only in juxta-position even in the most intimate plexuses." "*Ehrenberg* was the first to demonstrate by microscopical examinations that in every nervous bundle we may distinguish the motory from the sensory filaments; the former remaining after death quite cylindrical, and presenting only a slightly rugous surface, whereas the latter exhibit a distinctly varicose or nodulated appearance.

.... "Not only may we distinguish the sensory from the motory filaments, but we may also distinguish the filaments of those nerves belonging to organic life from those which belong to animal life: the former are of a red colour and extremely slender, whereas the latter are white and much more distinct."...... "Usually we observe in a nervous bundle the three sorts of primary filaments —the motory, the sensory, and the organic. The organic filaments, which proceed from ganglions backwards to the spinal marrow, become more and more slender, until they are at length lost in the substance of the cerebro-spinal axis."...... "In the cerebro-spinal system, there are two sets of actions performed; on the one hand, sensations perceived, and on the other, the reactions of volition. Two analogous actions are observed in the organic life; there is an organic perception, or what *Haller* called irritability; and there is a reaction, or the function of organic reflexion, so ably demonstrated by *Müller*. We may therefore say that in the animal economy there is a double *sensorium commune;* one, belonging to the life of relation, is the cerebro-spinal axis; and the other, belonging to organic life, is the ganglionic system."

---

### Fatal poisoning from Aconite.

On the 11th of June, twelve patients affected with scorbutus and pellagra were seized with extreme *malaise* about an hour after swallowing a dose of what was supposed to be the fresh juice of the *cochlearia*. One of them, a man of about sixty years of age, after suffering from dyspnœa, vomitings, and great anxiety, died in the course of a few hours. Two middle-aged women also, affected with pellagrous insanity, became restless, then convulsed, and at length paralytic, and died. It was supposed at first that they had an attack of mania.

The remaining nine patients fortunately recovered under the use of active treatment; they were all more or less affected with the following symptoms:—rapid prostration of physical and mental energy, dilatation of the pupils, distressing headache and vertigo, pain and tension of the abdomen, accompanied with borborygmi and vomiting of green-coloured matters, great anxiety and sense of oppression at the chest, coldness of the whole body but especially of the extremities, and livid colour of the nails, cramps in the legs, small and fluttering and occasionally imperceptible pulse, &c.—everything indicated a state of extreme hyposthenia or general prostration.

By the use of stimulants given inwardly, and of friction of the extremities with warm spirituous embrocations, these alarming symptoms were gradually removed. The *dissection* of the three persons, who died, revealed the following appearances:—

The vessels of the pia mater and of the arachnoid membrane were highly injected, and a small quantity of serosity was found between the meninges and the base of the brain. The lungs were gorged with blood; the heart was flabby and contained a small quantity of dark fluid blood. The mucous surface of the stomach and small intestines exhibited patches of vascular injection: the texture of the spleen was extremely soft. On making enquiries in the laboratory where the medicine had been prepared, it was found that the juice of the *cochlearia* had been inadvertently put into a vessel which contained some fresh juice of *aconite*, and that this mixture of the two had been dispensed to the patients. "This fact," says the reporter in conclusion, "while it is on the one hand deplorable by the loss of life which took place, is, on the other hand, very instructive; for it most distinctly confirms the accuracy of *Giacomini's* opinion, that aconite is to be regarded as a direct sedative or hyposthenic medicine, and that its proper antidotes are stimulants, such as spirituous liquors, opiates, cinnamon, &c. Some modern toxicologists have committed a serious error in regarding aconite as an acrid narcotic, which requires antiphlogistic remedies to counteract its stimulant effects. We observe no genuine traces of inflammatory action in the bodies of those who have fallen victims to this poison: the mere circumstance of patches here and there of vascular fullness along the alimentary canal proves nothing; for this appearance is observed in almost all cases where life is extinguished from the operation of sedative agents. The flaccid state of the heart, the softened lacerable condition of the spleen, the emptiness of the large blood-vessels, and the passive stasis of the blood in the substance of the lungs—all tend to prove that death was the result of a general prostration of the vital powers.—*Gazette des Hôpitaux.*

---

### M. Leuret on the Use of Belladonna in Epilepsy.

During the last three years, M. *Leuret,* physician of the Bicetre, has very extensively administered this potent medicine internally in cases of epilepsy, and the results of his practice have recently been published in the pages of the Gazette des Hôpitaux.

After detailing the reports of seventeen, out of numerous other cases treated with belladonna, the memoir concludes with the following observations:—

"If we now compare the results in these cases, we must see that, in almost every one, there was a decided amendment after the use of the remedy. This amendment was most conspicuous in the early period of the treatment; and the good effects seemed to be more lasting in proportion as the physiological effects of the belladonna were least marked. There was rarely an exception to this remark; in one case however there was certainly a striking coincidence in the cessation of the epileptic fits with the invasion of the almost maniacal delirium and agitation which followed the administration of the medicine. As a general remark, we may state that, in the majority of instances, it was rather in the diminution of the frequency than in the abridgment of the severity of the fits, that the amelioration was most decided. In some cases, however, the fits became not only much less frequent, but also much milder.

Another result of our observations is, that the effects of the internal use of belladonna on the general system usually do not remain energetic beyond a few days, even in cases where the doses have been considerable. Might it not therefore be prudent to alternate the use of this remedy with some other which is known to be useful in cases of epilepsy?

The effects of belladonna, internally administered, are most conspicuous on the circulation, the powers of vision, the nervous system, and the tongue.

1. In every one of our patients, the pulse was found from the second day of treatment to be accelerated: this acceleration continued for different periods of time, from three or four to fifteen or even twenty days, in different cases.

2. The same remark is applicable to the dilatation of the pupil; this having been observed in every case without exception. This effect was however more durable than the former one; it usually lasted for a length of time, and, in more than one case, during the whole course of the treatment.

Along with the dilated state of the pupil, there were, in some instances, confusion of vision, or partial blindness, myopia, &c.

3. The most remarkable of the nervous symptoms induced by the belladonna were a greater or less degree of restlessness and excitement, approaching sometimes to inordinate gaiety and at other times to furious delirium; frequent hallucinations; convulsive crying or laughing; a tottering in the gait, &c.

It is well worthy of especial notice that these nervous symptoms were always most decided in those cases, in which the disease resisted the use of the remedy. Should we consider them as the results of the disease rather than of the remedy?

4. One of the most constant and most durable of the effects of the belladonna is a very marked development of the papillæ of the tongue: there is frequently, at the same time, a sense of dryness of the mouth, and thirst. In no case, however, had we occasion to observe that feeling of burning heat in the mouth and throat, excessive thirst, and profuse diarrhœa, which some writers have attributed to the internal use of the remedy. In all our cases, the patients continued to take their food as usual.

With respect to the doses, in which we may administer belladonna, M. *Leuret* has, in almost every instance, been in the habit of ordering 30 centigrammes (about half a grain) at first, one-third part being given at equal intervals during the twenty-four hours, doubling this quantity on the second day, and on the fourth or fifth day raising it to 90 centigrammes, at least in some cases. By far the best preparation is the alcoholic extract; it may be given in the form either of pills or mixture.—*Gazette des Hôpitaux.*

*Remark.*—These observations, based as they are on the results of clinical experience during several successive years, deserve the notice of the practical physician.

We have no hesitation in saying that, in our opinion, the internal administration of sedatives is far too much neglected in the treatment of epilepsy and other allied affections of the nervous system. We have repeatedly seen excellent effects in such diseases from the exhibition of from ten to fifteen drops of the tincture of henbane three times daily for four, six, or eight weeks at a time —then omitted for a week or so, and again resumed in smaller and less frequent doses. Some of the metallic tonics, as the nitrate of silver, the sulphate of zinc, &c. either alone or in combination with quinine or valerian, may be advantageously given at the same time. In cases where a seton is deemed advisable, we have observed that it is often decidedly more useful if established over the heart than in the nape of the neck, as usually recommended. That epileptic and epileptiform diseases are, if not actually dependent upon, always accompanied with, a tendency to disturbances of the general circulation, must be known to every physician; and there is perhaps no remedy so efficacious against such disturbances as a permanent irritation in the cardiac region.—*Rev.*

### Cases of Neuralgia treated with Acupuncturation.

Case 1.—*Neuralgia of the Foot.*—A young woman, when 22 years of age, became affected with a most excruciating pain in the right foot, which was occasionally swollen and apparently highly congested. She was repeatedly bled and the foot was frequently leeched; but without effect. For ten months she was obliged to keep her bed. When admitted into the clinical wards of the Turin Hospital under the care of Professor *Riberi*, the seat of the pains and swelling was on the outer side of the foot; from this point the pains extended up along the leg, and the swelling affected the entire *dorsum* of the foot. Whenever the foot was lowered even a few lines below the horizontal level, the sufferings were much increased, and the same result followed the mere pinching of the skin. The "point de depart" of the pain was manifestly in the nerves of the foot. Antiphlogistic remedies were tried at first; but although the constitutional health, which had begun to suffer, was considerably improved under their use, there was no relief of the local neuropathy. M. *Riberi* then tried acupuncturation: ten needles were inserted into the flesh, and allowed to remain in for the space of an hour. The operation was repeated four or five days afterwards, and subsequently at seven different times. The second time, the needles were left in for two hours; then for three, four, and even five hours. From the date of the first operation, the pains were so much relieved that the patient could rest on the affected foot, and in the course of a month she was able to leave her bed and walk about, her sufferings having entirely ceased.

Case 2.—*Sacro-lumbar Neuralgia.*—Giuseppe Vola, 38 years of age, had in the course of the year been affected with hydrocele on both sides; he was radically cured in the one instance by incision, and in the other by injection of the sac with the tincture of iodine. Subsequently to this, he began to suffer from most severe sacro-lumbar neuralgia. "This I relieved as by enchantment," says Prof. *Riberi*, "by means of a single acupuncturation; the needles were left in for three hours. Three days subsequently the pain returned; and again it was as quickly relieved."

The place where the needles were inserted, and the number used, are not stated.

Case 3.—*Severe Brachial Neuralgia.*—A young woman, about 20 years of age, had been often affected with various nervous complaints. While being bled from the arm, she experienced, at the moment the incision was made, a violent involuntary trembling accompanied with an intense pain in the fold of the arm, and a rigid contraction of the fore-arm. This contraction continued with such violence that it could not be overcome by the use of splints or any other means that were used. When admitted into the hospital, the chief symptoms were the intense pain of the arm, complete loss of sleep, and considerable derangement of the digestive organs. The pain was of two kinds; the one dull and cramp-like, the other lancinating and burning. The seat of the former was chiefly in the course of the median nerve, and extended downwards from the fold of the arm to the fingers; whereas the latter, which was not accompanied with trembling of the affected parts, involved all the nerves of the arm, not excepting those of the skin, and sometimes passed on to the trunk, affecting the lungs and the heart: hence the sense of suffocation and of constriction of the chest, and the syncope, which often supervened during the paroxysms of suffering. The cicatrix presented a small, almost imperceptible, knot, which was certainly not the only, nor even the principal cause of the pain. The severity and continuance of the patient's sufferings at any time seemed to be much influenced by the state of the atmosphere.

The paroxysms of pain were brought on by either quickly extending or bending the fore-arm or all the fingers at one time, or by pinching the middle finger, or again by pressing on the cicatrix at the bend of the arm, or by gently rubbing the arm or fore-arm with the hand, &c.

Whatever was the exciting cause, the pain, developed either in the cicatrix or in the fingers, shot with the rapidity of lightning to every part of the limb, then to the shoulder, chest, and region of the heart, and the most frightful symptoms were induced.

To prevent all sort of friction, pressure, or extension of the limb, the patient, when walking lowered her shoulder and inclined her body to one side, as if she was affected with a lateral curvature of the spine.

After trying the effects of the internal use of belladonna and henbane, and of the external employment of prussic acid, &c., Signor *Riberi*, had recourse to acupuncturation. From ten to twenty slender needles were inserted into the flesh of the arm along the course of the median nerve, and allowed to remain in for from two to four hours. This operation was practised about twelve times in the course of two months. After each acupuncture, a very marked relief was obtained; and ultimately the patient was completely and permanently cured. It deserves to be mentioned, as a very striking proof of the efficacy of the remedy, that from the very first day of its employment the patient was able to move the arm without much distress, and the pain, from being fixed, became moveable, and ceased at those points where the needles had been inserted; so that by multiplying these punctures, the pain became more and more distant, and at length finally ceased. After the sixth operation, the patient did not complain of any pain, unless when firm pressure was made over the course of the median nerve or of its principal branches; and after the tenth operation, she was able to use her arm freely in her domestic occupations.

Case 4.—*Lumbo-sciatic Neuralgia.*—A countryman, 63 years of age, was admitted into the hospital with a large hydrocele of the right testicle. The urinary passages were excessively tender, and there was always more or less ischuria. The hydrocele had been preceded with severe pains not only in the affected testicle, but also in the right groin and in the loins, which had continued with great violence for ten days, and were followed by the occurrence of numerous varices of the veins in both legs, and more especially in the left one. Subsequently to this, the patient had suffered with symptoms of inflammation of the upper part of the spinal marrow—the chief symptoms were vertigo, stupor, spasmodic pains in the upper extremities, and afterwards a sense of dullness and weight in these parts. By the use of leeches, and of a seton, &c. these symptoms were entirely removed. The hydrocele, which was of several years standing, had acquired a considerable size; and the patient complained of intense neuralgic pains in the corresponding testicle, radiating from this point to the loins, and to the right sciatic region, in which it ultimately fixed.

After the evacuation of the water from the tunica vaginalis, the lumbo-ischiatic pain became more severe than ever, and the right limb was quite powerless. Twenty needles were inserted,—(it is not stated where)—and allowed to remain in for upwards of three hours. The operation was repeated four times; and a radical cure was obtained.

Case 5.—*Painful Paralysis of the Limbs.*—A middle-aged woman was attacked, subsequently to a troublesome disturbance of the menstrual function, with severe pains in the loins, which were gradually followed by a paralysis of both lower limbs. Every now and then she experienced spasms, which extended from the limbs to the abdominal parietes and spine, and were accompanied with formication, lancinating pains, a sense of tightness and constriction and of great heat. To these symptoms succeeded a numbness in the limbs, extreme sluggishness in the intestinal and urinary evacuations, and at length a state of complete paralysis. Various local stimulants had been tried; but they seemed to be rather hurtful than otherwise. This indeed might have been expected, seeing that the disease depended upon a sub-inflammatory state of the lower extremity of the spinal cord and of the principal nervous trunks which issue from this part. Under the use of perfect quietude and a mild antiphlogistic regimen, the patient somewhat improved; and Professor *Riberi* then had recourse to acupuncturation. Twenty needles were inserted into the sacro-lumbar region, and along the course of the sciatic nerves, and allowed to remain in for about three hours: this operation was repeated eight different times.

After the second operation, the pains had nearly ceased; and soon afterwards the bladder recovered its expulsive powers. At the end of three weeks from the first employment of the needles, the woman was able to leave her bed and walk about the wards without the aid of either crutch or stick. Gradually the limbs regained their healthy suppleness and force, and a complete and durable cure followed.

The last case reported by Professor *Riberi* was one of more than ordinarily severe lumbago, which had resisted all the usual remedies. Acupuncturation was therefore had recourse to, and with the best effects; for ultimately the patient was quite cured. The operation was repeated ten different times; eighteen or twenty needles being introduced each time. It was remarked that the pain never returned in the seat of the punctures.

The series of cases now detailed proves, in a very satisfactory manner, the powerfully remedial effects which the operation of acupuncture has in relieving various forms of neuralgic suffering. The attention of practitioners is apt to be distracted in the treatment of this too-often most unyielding complaint by the multitude of remedies which are daily proposed: acupuncture is certainly one of the most efficient, and deserves a more extensive trial than has hitherto been given to it.—*Gazette des Hôpitaux, No.* 96.

---

### Report on M. Petrequin's Method of Treating Deafness.

Since the publication of M. *Petrequin's* Memoir on the Treatment of Deafness by alum gargles and insufflations,* various experiments have been made in different parts of France and Belgium, to ascertain the effects of these remedies, and the Royal Academy of Medicine has directed its attention to the subject in the course of last year. A lengthened and very favourable report has been recently laid before the Medical Society of Lyons by M. *Brachet,* of which the following brief account may be interesting to our readers.

After alluding to M. *Petrequin's* opinion, that the use of the Eustachian tube is similar to that of the holes made in a drum—viz. to renew the air within the cavity of the tympanum—and presenting an abstract of the eleven cases reported at length in his memoir, the following important conclusions drawn by M. B. from his experience are given: 1. That deafness is of very frequent occurrence in old people, in whom the mucous membranes do not perform their functions healthily, and who are subject to catarrhal congestions; also in those persons who have been subject to any of the numerous forms of inflammation of the pharynx.

2. That the inspection of the throat furnishes a means of exploration which greatly facilitates the diagnosis, by often revealing a chronic and indolent inflammatory state of its mucous membrane.

---

* Vide Medico-Chirurgical Review for July 1839.

3. That Sir *A. Cooper* was mistaken in considering the existence of a humming noise in the ear as a symptom exclusively of nervous deafness; a form of the infirmity which is also of much less frequent occurrence than he supposed.*

4. That it is a serious error to consider as incurable all cases of deafness, in which the patient cannot hear the ticking of a watch placed between his teeth; and that it is highly probable that many persons are from this mistake left without medical assistance, which if applied in time might have relieved the infirmity.

5. That the diagnosis of obstruction of the Eustachian tube, easily established when redness of the pharynx exists, is much less so, when this has disappeared, and when the lesion of the tube alone remains.

M. *Petrequin*, however, is of opinion that we may suspect the existence of this lesion by the varying condition of the sense of hearing in different states of the weather, and when it is somewhat better, for a longer or shorter period of time, after a fit of coughing or sneezing.

6. That the prognosis or chance of cure in this form of deafness is by no means so unfavourable as *B. Bell* and Sir *A. Cooper* have alleged; that often it may be cured by fluid or gaseous injections, or by cauterization of the opening of the Eustachian tube in the throat, or by the use of alum applied either in the dry or liquid form to the back of the fauces—as a gargle, or as a powder mixed with sugar and insufflated upon the fauces, or lastly, the direct application of a stick of alum—recommended so strongly and used so successfully by M. *Petrequin*. He believes that this salt has a special effect on the pharyngeal membrane, independently of its merely astringent operation. However this may be, it would seem that it has a most beneficial influence on many maladies of the mouth and fauces. MM. *Bretonneau* and *Pommier* have strongly recommended it in the treatment of diptherite, Signor *Bennati* in various affections of the larynx, and M. *Velpeau* in diseases of the throat. Its administration is easy, and does not interfere with the employment at the same time of other remedies, as antiphlogistics, revulsives, purgatives, cauterisation, the catheterism of the Eustachian tube, and the baths of compressed air, which have been so successfully used by M. *Pravaz*.

Hitherto no trial has been made of injecting a weak solution of alum directly into the Eustachian tube; but it is more than probable that the practice will be found highly useful in many cases."—*Bulletin Medical Belge*.

*Remark.*—The reflections of M. *Petrequin*, based as they are upon the results of clinical experience, are certainly well deserving of attention. We should suggest the addition of tincture of capsicum to the alum gargle, as rendering it probably still more efficient in dissipating the chronic inflammatory state of the fauces and pharynx, which according to his observations, is so frequent an accompaniment of deafness.—*Rev.*

---

### ON SOME REMEDIES AGAINST SLEEPLESSNESS.

M. *Max. Simon*, after alluding to the various causes or states of the system that are apt to induce sleeplessness—in some of which it is merely one of many symptoms, and cannot therefore be relieved until the existing disease subsides,

---

* M. *Petrequin* is probably quite correct in his opinion, that the nervous form of deafness is not nearly so common as is generally imagined by medical men, and that the real cause of the infirmity in a great number of cases is a sub-inflammatory state of the mucous lining of the tympanum and of the Eustachian tube.

while in others it is an adjunct, or, as the French say, an epi-phenomenon, and may yield to remedies, although the disease remains unmitigated—proceeds to offer a few comments on some of the most valuable hypnotics, and on the best mode of employing them.

*Opium*, as a matter of course, is first on the list. From phthisis pulmonalis to cancer, and from hysteria to delirium tremens and tetanus, there are few morbid states of a non-phlogistic nature, in which sleeplessness may not give rise to a special therapeutic indication, and which may not be successfully combated by some preparation of opium.

An illustrious physician of the old school has said, that it is chiefly by the skill and tact displayed in the administration of this drug that one physician may be distinguished from another:—he was quite right; as no medicine requires more practical skill for its proper employment. "Sacra vitæ anchora circumspecté agentibus est opium; cymba Charontis in manu imperiti:" says *Wedel*.

How different are its effects on the system in different cases, where it fails of producing its narcotic action! For example, when administered in many cases of phthisis and of neuralgia, we often observe that, although it may not quiet the cough in the one case, nor the pain in the other, it does not give rise to any unpleasant symptoms either on the local or on the constitutional disease; its action seems to be merely neutralised and nullified. But it is far otherwise when the sleeplessness is the special symptom for which the opiate is given; for then, if it fails as a soporific, the agitation of the patient is singularly increased, and a general uneasy restless state, with headache and feverishness, is induced.

There was some reason, therefore, for *Brown* exclaiming, "*Mehercle, opium non sedat.*" We repeat, it is more especially when sleeplessness in any case is the predominant phenomenon that opium is apt to fail in procuring sleep, and to aggravate the nervous excitement which may be present.

The reason of this difference in the action of the same medicine in different circumstances seems to be that, when it is given to abate severe pain, or to quiet the cough in tuberculous affections of the lungs, the nervous system participates only very partially in the local morbid action; whereas the state of sleeplessness implies the existence of a morbid state which is altogether more complex, and which involves the whole nervous system. Hence it requires a much more rigid analysis of the general condition of the entire economy, to detect the phenomena which may compromise the soothing effects of the remedy. The pulse furnishes us with perhaps the most safe rules to guide our practice under such circumstances. If it be full and at all hard, the use of opium is contra-indicated; but when it is soft, compressible, and, so to speak, nervous, we may then anticipate that it will prove soothing and soporific.

Besides the usual methods of exhibiting opium, there is one which is much less frequently tried than it deserves to be;—we allude to the plan so strongly recommended by that veteran of the German school, *Hufeland*. This consists in the application of a plaster composed of henbane and opium to the temples.*

---

* The proportions recommended by *Hufeland* are, of the plaster of henbane half an ounce, and of that of opium a scruple. (Does he mean the extract of the two plants?) These doses may be varied according to the symptoms of the case; or the extract of belladonna may be substituted for that of the henbane. Either may be reduced to a syrupy consistence by the addition of a sufficient quantity of laudanum, and may then be applied to the temples by means of a portion of lint dipped in the mixture.

To ensure the better penetration of the skin, it is always well to cover the part when wetted with a piece of oil-skin; the absorption or imbibition is thus rendered more certain.

We have several times found that in this way sleep may be induced, without the risk of inducing any of the unpleasant symptoms which not unfrequently follow the internal use of all narcotic medicines. It is admirably suited for those cases in which sleeplessness has been brought on by intense grief, or by protracted application of the mind. The application should be repeated every evening, just before bed-time, for several successive nights. We do not know that a fair trial has been given to it in that not uncommon set of cases in which there is a most distressing excitement of the nervous powers, bordering almost on mental derangement; also in delirium tremens, incipient insanity, &c.

But it is not by *material* or physical means alone, that we may sometimes succeed in inducing sleep; the vagaries of the mind itself may be often used to summon back this " best balm of nature," when frighted from our couches.

Some German writers, as *Gruithuisen, Purkinje,* &c. have made a special study of dreams, and, by analysing their phenomena in a philosophical manner, they have been led to the conclusion that the various phantastic images of every shape, colour and size, which are so often seen to dance before the eyes when we close them for sleep, are veritable elements of dreams.

Now *Burdach,* availing himself of this idea for therapeutic purposes, suggests that, as these fantastic images are the beginnings, as it were, of a dream, they will necessarily induce sleep, if we can but tranquillize ourselves sufficiently to regard them, and contemplate their play without any intellectual reflection. The experiment is easily made by every one; and we have often satisfied ourselves of the correctness of *Burdach's* suggestion.

We must not, be it remembered, endeavour at the time to analyse and explain the phenomena, but only keep our attention fixed upon the fantastic image, without doing more. Gradually it (the image) becomes fainter and more indistinct, until a complete dreamy state with all its faëry scenes comes on:—now, *to dream, is to sleep.*

Some other writers, who have paid much attention to this curious department of physico-mental study, have suggested, as a useful means of inducing sleep, that the attention should be kept fixed upon the mental image of a calm unruffled sea, or on that of a vast desert, in which there is nothing to break the wide expanse of uniformity. It is this very uniformity of the image, pictured by the imagination, that brings on a drowsy unimpassioned state of feeling which is akin to sleep.

M. *Simon* would seem to be a partial believer in the remedial powers of magnetism, (we suppose animal magnetism); for he suggests that a trial of it should be made in certain cases of sleeplessness, in which the use of opiates, &c. either has proved ineffectual, or may be deemed inexpedient. He mentions the case of a lady, suffering from all the tortures of cancerous disease of the uterus, in whom he witnessed the soothing and soporific effects of this agency. But he is a timid disciple of Mesmerism: for he candidly confesses that "the fear of acquiring the name of a magnetiser prevented him from repeating his experiments in the case alluded to more than twice or thrice!" (We need scarcely say that whatever withdraws the attention from a local suffering, and keeps it fixed on any mental exertion, will often have the effect of making us entirely insensible to pain. How often do we read of soldiers and sailors, during the hurry and excitement of action, being actually not even aware that they have sustained some frightful wound for some time afterwards? and who has not experienced in his own case that many a sharp ache has been charmed away by the report of some unexpected news, by the lively conversation of a friend, by the perusal of a fascinating book, or by the mere repetition to oneself of various passages from some favourite author? There is therapeutic as well as moral wisdom in old Spencer, when he says,—

> " It is the mind that maketh good or ill,
> The wretch or happy............ "

We should therefore be cautious before admitting the existence of any special agency, such as the animal magnetisers seek to prove; although we must, at the same time, confess that it is far from improbable that living animal bodies may have some modes of operation, one upon the other, which have hitherto escaped our exact scrutiny.—*Rev.*)

M. *Simon,* in concluding his observations, alludes to that sleeplessness which is not unfrequently noticed at the decline of inflammatory and other acute diseases, and very properly says that a restless state of the nervous system is apt to be induced by pushing antiphlogistic measures too far, or by continuing their use beyond a certain period; the cautious use of gentle tonics, and more especially of light food, being in general the best remedy in allaying irritation and inducing sleep.—*Bulletin de Therapeutique.*

---

### Treatment of Small-pox with Mercurial Applications.

It is scarcely necessary to remark that the *ectrotic method,*—which consists in the application of the nitrate of silver to the pustules—of treating small-pox was proposed some years ago by M. *Serres* as a most effectual means not only of preventing the pitting of the surface, but also of rendering the constitutional disturbance less severe. It has been fairly tried by MM. *Velpeau* and *Bretonneau,* as well as by many other medical men; and their experience has been on the whole unfavorable to its adoption. The chief objections seem to be the pain which is caused, and the great difficulty of applying the caustic to all the pustules.

Subsequently M. *Serres* has strongly recommended the application of the *emplatre de vigo,* a mercurial ointment or plaster, to variolous pustules; and we are pleased to find that the experience of several other good observers has fully confirmed the favorable reports made by him of this practice. In the late epidemic of small-pox in Paris, Dr. *Briquet* gave an extensive trial to the mercurial inunction, more especially of the face and the other exposed parts of the body; and the result of his observations is strongly in favour of M. *Serres'* proposal. Within the last few months, M. *Chomel* also has been adopting it in his practice at the Hôtel Dieu; and the following cases are drawn from his clinique.

*Case* 1.—A girl, 19 years of age, was admitted with all the symptoms of regular semi-confluent small-pox. A mask made with the *emplatre de vigo* was applied over the face on the second day of the eruption. Although it was torn off by the patient four and twenty hours afterwards, its effects seemed to be very remarkable. Whereas the pustules on the neck, chest, and body exhibited all the usual characters of genuine small-pox, the eruption on the face appeared to be quite abortive, being either merely vesicular, or altogether solid and papular. Wherever the plaster had been applied, such was the character of the pock; but in every other part it was distinct and full-formed.

*Case* 2.—In the St. Augustine ward there is another variolous patient, who has been treated in the same manner, and who is now convalescent. The desquamation has followed its usual course, except on the face, where scarcely any scabs have formed, and then only in those points which had not been acted upon by the plaster. This case is interesting, as it confirms the assertion of M. *Serres,* that there is no desquamation under the influence of the mercurial plaster.

*Case* 3.—In the same ward there is a woman, who is in the sixth month of pregnancy, and is labouring under well-marked small-pox. M. *Chomel* did not hesitate to apply the mercurial plaster to the face; and the inspection of the patient will shew how complete the success has been. The form of the eruption has exhibited in this patient some peculiarities. A semicircular zone or band of a bright red colour, and about two inches broad, extended from over the lumbar vertebræ to the axilla of either side: the redness did not disappear on pressure. The temperature of this zone was greater than that of the rest of the body. At first it seemed to be different from the general malady; but soon there were developed over the course of the zone distinct, but extremely small, and agglomerated variolous pustules.

The eruption on the face became quite abortive, the pustules remaining small and of a whitish appearance; there was no scabbing, excepting at the edges of the lips and the eyelids, where the action of the mercurial mask had not taken effect.

These cases appear to confirm the opinion of M. *Serres,* that the mercurial application to the pustules of small-pox has a most powerful influence in modifying their usual course; the pustules so treated being not surrounded with their usual red areola, the suppuration being very imperfect, and the swelling and tension of the integuments being trifling or altogether absent.—*Gazette des Hôpitaux.*

*Remark.*—The circumstance of this practice being favourably reported of by so experienced and cautious a physician as M. *Chomel,* gives us a warrant to try it fairly on this side of the Channel.—*Rev.*

---

### M. Lugol on Cutaneous Scrofula.

A few extracts from some of the lectures recently delivered by this distinguished physician will be read with interest.

. . . . . . . . " Scrofula of the skin sometimes commences with a simple fissure or crack, which causes a most troublesome itching. A crust or scab forms upon the part; and, when this falls off, we often find an ulcerated surface of greater or less depth and extent.

At other times, the disease begins with a red patch; the skin becomes thickened and indurated; it assumes a livid aspect, and pustules form upon it—in other words, there is an eruption of the pustular form. Occasionally the pustules are excessively solid and hard, and are then improperly termed tubercles; —improperly we say, because they always terminate in suppuration, and then exhibit no difference from pustules.

In a third set of cases, cutaneous scrofula commences by the generation of a tuberculous deposit under the skin, which becomes in consequence elevated and detached. In course of time, this becomes reddened and eventually gives way; and the tuberculous matter is then found transformed into a genuine pustule.

Cutaneous scrofula most frequently is seen in the teguments of the face; very often on the alæ nasi, the cheeks, chin, and eyelids. It is much more rare on the neck, body, and extremities."

. . . . . . . . "The disease necessarily involves a derangement of the functions of the skin. One of the most remarkable symptoms is an increase in the perspiration of certain parts, especially the axillæ, hands, and feet. There is established a sort of supplementary function, which becomes a *besoin,* and an indispensable condition of health. I often see a young girl in whom this abundant secretion of the transpiration is almost constantly present, and who is at the same time affected with leucorrhœa.

If this excessive discharge of the perspiration is suddenly checked, the health often suffers; a troublesome cough, for example, and a state of general malaise is induced; and these symptoms will sometimes not yield, until the checked secretion is restored."

*Frequent Co-existence of Lice with Cutaneous Scrofula.*

"The greater number of the scrofulous patients in this hospital are affected with them; and for many years past I have had many opportunities of pointing out this curious coincidence to my pupils. It would be quite erroneous to refer this to any want of cleanliness on the part of the patients; for they are all obliged to attend to their persons with the greatest care; and moreover we not unfrequently observe these vermin in scrofulous children of the higher classes, who are invariably kept with the greatest nicety.

Another sign, or perhaps more properly speaking, another frequent complication of scrofula, is the tendency to severe chilblains; whenever these are very obstinate, you may suspect the person's constitution.

In some cases, the skin is unusually dry and subject to pruriginous eruptions; in others, it is generally moist and unctuous.

It is of great practical importance to ascertain the constitution of a patient affected with any cutaneous disease; for all the forms of it are invariably more severe and unyielding in persons who are scrofulous. How often do we find that, when the eruption on the skin is removed, an ophthalmia, or leucorrhœa, &c. makes its appearance, and resists for a great length of time all remedial means. When some of the forms of impetigo occur, the cuticle, not only of the pustules themselves but of the entire extremity, or of the face if it happens to be effected, separates, leaving an immense excoriated surface. I lately had a young man under my care, in whom both arms were completely denuded of their epidermis, and a great part of the lower extremities at the same time. It is interesting to watch the reproduction of the cuticle in such cases: various islands, as it were, are formed in different parts, and these gradually widen more and more until they coalesce, and the entire surface is re-covered with its epidermal investment.".......... *Gazette des Hôpitaux.*

---

### Two Cases of Spontaneous Emphysema.

The first case occurred during the act of parturition, and the patient gradually recovered.

A woman, 28 years of age, and who had never suffered from any affection of the respiratory organs, suddenly during the pains of childbirth, lost her voice, and at the same time felt a swelling on the fore part of the neck; this gradually extended over the front of the chest, the sides of the neck and the face.

The integuments were so much puffed out, especially on the face, that the patient could hardly be recognised by her attendants; when pressed upon, a distinct sound of crepitation was audible. The patient felt a good deal oppressed at the chest, and complained of a sense of tingling over the skin. As the labor was very tedious, the child was delivered by means of the forceps. The emphysema continued with but little diminution for four or five days, and then gradually subsided; but for some time afterwards the crepitation was perceptible in the parts which had been inflated.

This species of emphysema is not unfrequently observed to take place after any violent exertion, in consequence, no doubt, of the person forcibly holding his breath, while the lungs are more than usually filled with air. The air must escape from the upper part of one of the superior lobes of the lungs, as the emphysematous swelling almost always occurs at first about the lower part of the neck. Usually all that is necessary to be done is to keep the patient perfectly quiet and silent.

The *second* case was more serious.

A young girl had been ailing, for five or six months, with symptoms which were attributed to the approach of menstruation. She suffered at the same time from a certain degree of difficulty in swallowing, and an uneasy feeling in her throat; but these symptoms, although constant, were considered by her medical attendant as of an hysterical nature.

On the 20th of March, however, she became decidedly worse; she complained of severe pain in the throat, and an utter inability to swallow any solid food; the jaws could not be separated but with the greatest difficulty. For the next three days, she continued in nearly the same state; but, on the evening of the 23rd, the face and neck became suddenly emphysematous. Every time that she cried, the distention became more considerable. Next day, it had extended over both arms, the chest and abdomen; several scarifications were made in different parts, but the air was found to accumulate as quickly as it escaped. On the evening of the 25th she died.

*Dissection.*—No lesion could be detected at any point of the lungs; but on examining the larynx and trachea, a minute ulcerated opening was observed in the right ventricle, immediately below the vocal chord. Four or five superficial ulcers also were found on the pharynx.

Might not the operation of tracheotomy have been useful in such a case? The difficulty is in diagnosticating the seat of the rupture, whence the air has escaped into the cellular tissue.—*Gazette Medicale.*

---

### ON THE DISEASES INDUCED BY MERCURY.

Dr. *Dieterich,* of Munich, has published a lengthened work on the numerous forms of mercurial disease, in which he treats at length of all the maladies which are, or are said to be, induced by an excessive or imprudent use of this mineral—as hydrargyria or mercurial fever, salivation, pancreatic ptyalism, diabetes, hydrosis or excessive perspiration, various exanthemata, as eczema, herpes, miliria, &c. different forms of ophthalmia, angina, periostitis, enlargement of the lymphatic and also of the parenchymatous glands, ulceration of the mucous membranes, neuralgia, asthma, tremors, paralysis, and several other diseases, including a peculiar form of cachexia.

We have no intention of following our worthy author through his somewhat lengthy lucubrations. We shall select only a few excerpta.

In the *treatment* of *salivation* he recommends, besides the use of gentle aperients, sudorifics and cooling astringent gargles, the internal administration of iodine and creosote, both of which remedies have, he thinks, a marked effect in giving tone to the weakened salivary glands. The formula in which he prescribes the latter medicine is as follows:—

℞. Olei Creosoti ʒss.

Pulv. Lycopodii ʒij. Misce.

Divide into sixty pills, of which three are to be taken twice a day at first, and the dose to be gradually raised to five three times in twenty-four hours.

The following are *said* to be the symptoms, &c. of *Mercurial affection of the Pancreas.*

"The pancreatic mercurial ptyalism, or alvine sialorhœa, has hitherto been generally confounded with the mercurial diarrhœa, which often accompanies it: it may, however, exist alone. It is indicated by a pain in the left hypochondrium which gradually extends towards the epigastric region, by unpleasant eructations, and a purging of white or greenish serous frothy matters. The eyes are languid, the face is pale, and the tongue dry; there is a most unpleasant, often metallic, taste in the mouth; the thirst is great, the skin cold, and the pulse small and rapid; subsequently, the colicy pains become more severe, and the region of the pancreas is the seat of a burning pain, increased by the slightest pressure, &c. &c. The best treatment for the relief of these symptoms is to cause a derivation to the surface of the skin by the use of hot baths and epispastics, and to soothe the local suffering by administering some opiate preparation. Subsequently the use of mild astringents, as calumba, or the Peruvian balsam, is decidedly useful.

The acetate of lead promises to be of considerable benefit; and so does iodine, provided no inflammatory symptoms be present. Tonic and analeptic medicines will be required to restore the general health."

*Mercurial Miliaria* is described to be a much more serious disease than is generally imagined.

........ "This form of miliary eruption is connected with a striking disturbance of the nervous system. An imperfect pyrexia, attended with a most distressing sense of inward anxiety, and sometimes with more alarming symptoms, such as great restlessness, delirium, and even convulsions, precede its appearance. The pulse is usually small, soft and compressible; the urine is very pale; and the skin is bedewed with a faint-smelling perspiration.

Often the eruption disappears for a time and then returns; and sometimes this alternate retrocession and re-appearance take place repeatedly. This form of mercurialism usually terminates fatally; death being attributable either to an extreme exhaustion of the nervous energies, or to a dangerous pulmonary congestion being induced during the retrocession of the eruption. The treatment of such a case should consist in endeavouring to cause a derivation of the morbid action to the skin, in counteracting the alteration of the blood, and in relieving the nervous system."

*Mercurial Ulcers*........ "These usually form on the mucous membrane of the mouth or nose. There is observed a dark-red or livid spot, which becomes white, then ulcerates and discharges at first a thickish grey-coloured sanies, and afterwards a thin ichor, giving to the mouth a fœtid smell which is easily recognised.

The ulcer gradually enlarges, creeping along the gums and inner surface of the cheek; it almost always gains more in extent of surface than of depth. It bleeds upon the slightest touch, and the pain is often most excruciating when any thing is applied to it. Sometimes we observe that a distinctly venereal sore changes its nature, and assumes the mercurial character. When such is the case, it becomes first encircled with a livid circle; the edges swell; small bloodvessels are often seen creeping along its surface; and the purulent discharge becomes of a thinner consistence.

Whenever such a change takes place, the use of mercury should be at once discontinued; opiate and mucilaginous applications are best at first, and subsequently aromatic fomentations and gargles; steel, mineral acids, &c. will be required to repair the health of the patient."

........ "*Mercurial Neuralgia* is usually erratic, and not fixed in one part; sometimes it shifts about along the course of one nerve, and at other times it passes quickly from one nerve to another. It has distinct, but generally irregular, periods of intermission. The pain is always aggravated in damp weather, and by the application of wet or moisture to the affected part; and the patient finds himself easier in warm dry weather and in bed.

It would seem that this form of neuralgia is very much affected by the electrical conditions of the atmosphere.

By far the best remedies are sedatives, and ferruginous preparations.". . . . . —*Journal des Connois. Med. Chir. Juillet.*

---

### ON THE SEAT OF GONORRHŒA IN WOMEN.

In the last Number of the *Medico-Chirurgical Review,* page 207, there is a notice of M. *Gibert's* opinions on this subject. He maintains that " the seat of election of the disease in women, to use his own phrase, is the meatus urinarius, as in man; and that in the majority of cases the vagina is little, if at all affected, but that the cervix uteri very generally is so when the disease continues."

Although the correctness of these statements may be questioned by many, it is admitted by all the best writers on gonorrhœa in women, that the chief seat of the discharge varies a good deal in different cases.

Old *Astruc* described four forms of the disease. . . . . . . " The *first* occupies the *prostate,* which in women embraces the urethra and opens into the vulva under the clitoris on each side of the urethral orifice; the *second* affects the glands of *Cowper,* situated in the perineum near the anus, which open at the side of the carunculæ myrtiformes; the *third* affects the botryform glands on the surface of the vagina (vaginitis); and the *fourth* the cells on the surface of the urethra (urethritis)."

*Bosquillon* also, the translator of B. Bell's work on the venereal disease, admits four varieties of the disease, which, he says, is—1, either limited to the inferior part of the vagina;—2, or is seated in the mucous glands which surround the orifice of the urethra, and whose excretory ducts open on the membranous plane which extends from the clitoris to the superior arch of the vaginal opening;—3, or affects the urethra itself;—or 4, the numerous glands with which the larger and smaller labia are supplied.

M. *Ricord,* while he admits that he has not been able to discover any exact relation between the special nature and the particular seat of the disease, says that, " whatever may be the cause of the discharge, the vulva, the urethra, the vagina, and the uterus, may be either separately or simultaneously affected."

It may be useful to offer a very few remarks upon these four forms—viz: *urethritis, vulvitis, vaginitis,* and *uteritis*—which, be it remembered, are often co-existent in the same case, but which occasionally are observed separate and uncombined.

*Blenorrhagia Urethralis* (*Urethritis*).—Much difference of opinion has existed among authors as to the frequency of this form. *Swediaur* says that it is only the orifice of the canal which is inflamed in gonorrhœa, and tells us that he had never seen a single case in which the urethra was the seat of the disease. *B. Bell* however admits that the discharge sometimes comes from the canal of the urethra. *Bosquillon* has seen cases in which a urethritis continued a long time without any cotemporaneous affection of the vagina. . . . . . . . . " It is not uncommon to observe this form of gonorrhœa engender an inflammation of the vagina." M. *Cullerier* however seems to be of a different opinion: according to him, by far the most frequent seat of the discharge is the vagina, and the pus, which seems to come from the urethra, is only deposited on its surface.

M. *Ph. Boyer,* although he admits that *in all cases* the urethra is affected, says, that as the chief phenomena take place in the vagina, in consequence of its extensive surface, and as the inflammation of the urethra is observed only in the early stage, he prefers the term *vaginitis,* to that of *urethro-vaginitis,* to denominate the disease.

On the other hand, M. *Ricord* tells us, as the result of his experience, that urethritis is so frequent, that he should calculate it to be present in eight out of every twelve cases of gonorrhœa in women caused by impure connexion.

This statement is certainly not warranted by our own observations. We should rather say that it is only in rare and exceptional cases that urethritis occurs primarily and independently of any affection of the vagina and the other neighbouring structures; and that these parts are very frequently the seat of the disease, while the urethra is little, if at all, affected.

When the urethra in women is inflamed, the ardor urinæ is often more severe than it is usually in men, and the bladder is apt to be very soon affected in consequence, no doubt, of the shortness of the urinary canal in the former.

*Blenorrhagia Vulvaris, (Vulvitis.)*—Of all the forms of the disease, this is attended with most suffering: hence, whenever a woman complains of severe pain in the parts, dreads the passage of the urine, and walks with difficulty and distress, we may be sure, before examining her, that the blenorrhagia is seated in the vulva. It would seem to be the case sometimes, according to the experience of *John Hunter* and others, that all the symptoms of vulvar blenorrhagia may be present, and yet the parts, although tender on the slightest pressure, do not exhibit, on examination, the usual appearances of inflammation. The discharge is occasionally so very acrid that it excoriates not only the external genital organs, but also the inside of the thighs, if it come in contact with them. This is most frequently observed when there are numerous flat tubercles on the vulva—a very common complication of this form of blenorrhagia.

When the inflammation of the vulva is intense, it is apt to become deep-seated and œdematous. An abscess sometimes forms in the substance of the exterior or interior labia, and is apt to leave a most troublesome fistula. The caruncolous opening of the urethra is generally inflamed in all cases of vulvitis; but its canal is not necessarily affected. The same may be said in relation to the vagina.

The blenorrhagia of children and of young girls, who have never been exposed to any sexual contagion, is always *vulvar*, and seldom extends inwards, either along the urethra or vagina. We may remark that a very obstinate form of the disease is apt to be induced by onanism.

*Blenorrhagia Vaginalis (Vaginitis.)*—This is certainly one of the most common forms of the disease in females.

In certain cases—and this circumstance deserves especial notice—the inflammation does not affect the outer portion of the canal, but only its upper or interior half. We must not, therefore, conclude in any case that there is no vaginitis, merely because the external genital parts are not at all inflamed. It has been, no doubt, in cases of this sort that *Hunter* has seen the disease communicated to men by patients, who did not exhibit the slightest outward mark of being infected.

No form of the disease is so prone to pass into the chronic state, as the vaginal: in spite of the most judicious treatment it will often prove most obstinate, and last for many months. In a vast number of the cases discharged from a hospital as cured, the disease returns soon after, and thus the infection is more and more extensively propagated.

*Blenorrhagia Uterina (Uteritis Colli).*—When the disease affects the uterus, it is always limited to the neck of the organ. The inflammation is usually superficial, and seldom produces much tumefaction of the part. Writers differ a good deal as to whether the cervix is generally more sensitive than in health, or not:—some say one thing, and some another. The exudation from its surface is usually very viscid; it is either nearly transparent, or it is opaque and puriform.

The disease is apt to be especially obstinate during pregnancy, and often will resist every remedy that is tried, until after delivery takes place.—*Journal des Connois. Medicales.*

---

### TREATMENT OF GONORRHŒA WITH CUBEBS AND ALUM.

A correspondent in the Journal des Connoissances Medicales recommends very highly a combination of powdered cubebs and alum in the treatment of gonorrhœa, and adduces several cases in illustration of its efficacy. The formula, which he uses, is the following:—two ounces of cubebs and half an ounce of powdered alum are to be mixed well together and divided into nine doses, of which one is to be taken three times in the course of twenty-four hours.

A cure is said to be usually effected in from six to eight days.

*Remark.*—It is more than probable that alum has not been used in cases of gonorrhœa so much as it deserves to be. That the salt is rapidly absorbed into the system, and is eliminated, in a great measure, by the urine, appears from numerous experiments.

---

### APHORISMS OF PRACTICAL SURGERY, FROM DUPUYTREN'S LECTURES.

Dr. *Bigal,* a pupil of the late Surgical *Chef* of France from the year 1818 to 1822, has published a series of aphorisms drawn from the lectures delivered by him at the Hôtel Dieu. They amount to ninety; we shall select what seem to us to be the most valuable.

1. When the tibia and fibula are fractured at the same time, the seat of the fractures of the two bones is never at the same point.

2. The fracture of the upper part of the fibula is always a direct fracture, and is never produced by a *contre-coup,* as *Pouteau* has asserted. The patients may be able to walk about immediately after the accident. It differs from fracture of the lower part of the bone, both in its producing cause, in the absence of displacement of the fragments, and, lastly, in its mode of treatment, as nothing is required for the cure but rest.

3. Fracture of the lower end of the radius is often mistaken for luxation of the carpus backwards, and the true nature of the accident is not discovered during the formation of the callus. It is then found that the carpus projects backwards, and the end of the radius forwards; that the extremity of the ulna projects towards the inner side of the fore-arm; that there is a sinking in of the radius, as if it had been cleft with a hatchet; and that the inter-osseous space, so necessary to the movements of rotation, is effaced.

4. Surgeons are very apt to commit mistakes in their diagnosis of the different fractures to which the fore-arm is liable; and yet it is most necessary for the judicious treatment of each, to have formed an accurate opinion of what accident has taken place. The most frequent fracture of the fore-arm is that of the radius alone; next that of the two bones together; and lastly, that of the ulna alone. In the treatment of fractures of the fore-arm, it is always proper to place two graduated compresses, one on the palmar and the other on the dorsal surface of the limb, and also two splints, and a roller to be passed circularly round: this bandage has the advantage of keeping the two bones apart and of maintaining the inter-osseous space.

5. A fracture of the patella is never united by a perfectly formed callus within eighty days or so. The provisional callus, which exists at the end of about thirty days in other fractures, is not sufficient here.

6. What renders the consolidation of fractures of the patella difficult, is that the fibrous tissue, which is necessary to the formation of the definitive callus, exists on the anterior surface only, and not on the posterior surface, of this bone. The neck of the thigh-bone is nearly in the same condition.

7. Whenever, after forty or fifty days of the treatment of a fracture, the callus becomes painful, we have reason to fear that it either has given way, or is about to give way, and that the limb will become deformed.

8. Hæmorrhage from the ear, accompanied with coma, almost invariably indicates a fracture of the base of the skull.

9. Dislocations of the phalanges are usually very difficult to be reduced; much more so than those of large joints. The cause of this may be, that the lateral ligaments remain entire; but *Dupuytren* was of opinion that it was attributable chiefly to the displacement of the tendons, and their escape from the grooves in which they play.

10. There is one sort of luxation of the shoulder, which is exceedingly difficult of reduction; viz. that in which the head of the humerus is directed inwards and upwards, and which is usually occasioned by a fall down a staircase. The displacement is to a considerable extent; the head of the bone touching the clavicle, and being situated above the level of the coracoid process.

*Dupuytren* determined by numerous experiments on the dead body that the main obstacle to the reduction is that the beak of this process is often entangled in the substance of some tendon or muscle; when such is the case, no mechanical effort can overcome the resistance without danger.

10. Various accidents may arise from falling with force upon the feet; as, for example, fracture of the heads of several of the metatarsal bones, fracture of the os calcis, rupture of the vault of the foot in consequence of the ligaments being lacerated, luxation of the astragalus, and comminuted fracture of the tarsal extremities of the tibia and fibula.

11. No disease is more difficult of cure than paralysis of the arm induced by dislocation of the humerus. The paralysis seems to arise from the stretching, compression, and perhaps also partial rupture of the nerves, which form the brachial plexus. Often no remedial means are of any avail.

12. Congenital ruptures present this peculiarity, that the seat of their strangulation is most frequently in the neck of the herniary sack, and not at the ring. *Wilmer* has made this remark; and *Alanson* also has observed, that almost all the cases, in which the stricture is situated in the neck of the sac, are cases of congenital hernia.

13. The strangulation at the orifice of the herniary sac is very common, whereas it rarely takes place at the orifice of the ring;—this opinion is not shared by all authors on the subject. (Indeed the very opposite doctrine is maintained, we believe, by many surgeons. Were *Dupuytren* right, the operation of dividing the ring without opening the sac would be almost invariably fruitless.—*Rev.*)

14. Whenever vomiting ceases during the inflammation occurring in cases of hernia, we may be almost assured that the intestine has become gangrenous.

15. There are few patients so apathetic and *insoucians* as those affected with diseases of the urinary passages. (We should not have thought that; urinary and rectal diseases have usually appeared to us to give rise to more than ordinary anxiety and depression. This is often the case in *renal* disease.—*Rev.*)

16. Few diseases are more difficult to cure *radically* than a very tight (tres grande) stricture of the urethra. For, after the canal has been widened by the prolonged use of bougies, there is always a great tendency to a relapse of the disease. It is then that cauterisation becomes useful, because we thus obtain a cicatrix *moulded* upon the bougie.

17. There are cases of stricture, &c. in which the keeping of an instrument in the urethra, instead of being a means of cure, becomes actually an obstacle to it: *Dupuytren* used to cite several instances of urinary fistulæ cured by the mere withdrawal of the sound.

18. All the diseases, which proceed from contraction of the urethra, are almost invariably the result of previous attacks of gonorrhœa. The size and force of the stream of urine gradually become less and less; then it escapes only in drops, and at length there is perhaps a complete retention—a state that is usually followed either by paralysis of the bladder, or by rupture of the urethra at some point, and the effusion of the urine into the cellular substance of the perineum.

19. We frequently meet with abscesses about the anus or in the perineum in phthisical patients; and it is often dangerous under such circumstances to operate, as the thoracic symptoms are very apt to increase, when the local disease is meddled with.

20. Ulcerations situated between the toes are usually very difficult to heal; this seems to be owing to the lodgment of the discharge, the admixture of the perspirable matter with it, and the constant contact of the ulcerated surfaces.

21. Of all cases of caries, the most dangerous are those in which the sternum is affected; for, when once the spongy texture of this bone becomes diseased, very troublesome fistulæ are formed, and the patient generally sinks under the effects of the disease.

22. Caries of the crest of the os ilii is a not unfrequent cause of symptomatic abscess in the lumbar and sacral regions.

23. It is a fact of almost constant occurrence, that diseases of the upper part of the thigh are felt, so to speak, at the knee, and also that those of the upper part of the humerus are felt at the elbow.

24. After amputation of the limbs, affections of the chest often supervene. Whenever we have cause to apprehend this occurrence, we should have recourse to blisters over the chest.

25. It is a curious circumstance that, in certain individuals, after lithotomy or other great operations, an abscess is apt to be formed in the calf of the leg: we cannot form any idea how this should be; but so it is.

26. In hospitals we often observe cases in which a succession of abscesses, in almost every part of the body, takes place, without any previous local or general inflammation. Such cases surely afford a proof of a purulent diathesis of the system.

27. Ambulatory or erratic erysipelas usually terminates in the formation of abscesses. These abscesses generally form without pain, and often without the patient being at all aware of their development. Such an occurrence is too frequently the image and counterpart of what is going on in some internal part; a slow inflammatory action is set up and terminates in suppuration, without either pain, fever, or any outward symptom being manifested.

28. It is a well-known fact that all abscesses caused by small-pox exist between the periosteum and the bone, with tumefaction of the latter, and subsequent formation of a sequestrum; but, in the majority of cases, this cause produces only a swelling of the bone with denudation. It is important to distinguish these two sets of cases.

29. Syphilitic exostoses do not always disappear, although their primary cause has been entirely removed.

30. The sudden extension of the fingers, when they have been long bent, (in consequence, for example, of the contraction of the cicatrised integuments after a burn,) is not unfrequently followed by gangrene. The extension should therefore be slow and gradual; and we should avoid dividing or excising the bridle, caused by the contracted cicatrix.

31. It is not prudent to divide the frænum for phymosis during the existence of a gonorrhœal discharge, as the wound is then apt to degenerate into a troublesome ulcer.

32. In all diseases of the neck of the uterus, the posterior lip of the os tincæ is more deeply affected than the anterior one.

33. In general, in affections of the brain, the effects of purgatives on the bowels are much less powerful than usual: for example, five or six grains of tartar emetic, and several ounces of Epsom salts, will often not produce either vomiting or purging. In these cases the oleaginous purgatives, as castor oil, croton oil, &c. succeed best.

34. Hiccup, occurring in the course of diseases, is usually only a nervous complaint. Shivering is a much more dangerous symptom; it generally indicates the development of some internal mischief.

35. Patients, suffering from extensive and severe burns, have almost always a very constipated state of bowels. We should not be too anxious to remove this state, as it does not seem to give rise to any inconvenience; and, when strong purgatives are used, a most troublesome diarrhœa often ensues.

36. In the majority of cases of fatal burns, the internal surface of the stomach and of the intestinal canal is found to be highly injected. In the treatment therefore of severe injuries of this sort, the surgeon's attention should be directed to the condition of these parts.

37. Very severe burns often induce fatal tetanic symptoms.

38. Enemata with laudanum are the best means that we can employ to relieve the accidental and transitory delirium, which often accompanies surgical diseases.

39. When a cataract forms in youth, it is almost always in the membrane of the lens; whereas in old age it is the substance of the lens itself that becomes opaque.—*Journal des Connoissances Medico-Chirurgicales.*

---

### Memoranda from M. Lisfranc's Surgical Clinique.

*If fistulæ produce the indurations which surround them, these indurations have also the effect of keeping up the fistulæ.*

In the treatment of an old callous ulcer of the leg, you will find great difficulty in effecting a cure, until you bring into a healthy condition the surrounding indurated tissues; a fistula, accompanied with such a state of parts, is still more difficult to manage.

One of our patients had a number of deep fistulæ on his left thigh, which was indurated and nearly double of its usual size; every attempt at effecting a cure had failed; and one surgeon had even proposed amputation at the hip-joint. I directed my attention to the removal of the existing engorgement; the inflammatory element was present there; I therefore employed local bleedings and emollient poultices; and, when the inflammation was dissipated, the ioduret of lead ointment was applied. Under this mode of treatment, several of the fistulæ healed up. Compression, which had formerly failed, was now resorted to with such excellent effects, that in the course of three months the patient was entirely cured. I could easily adduce a great number of other similar cases to demonstrate the utility of the principles which I have so often explained—get rid first of the local inflammation, before you use any means to induce cicatrisation. If a fistula remains after all the inflammatory symptoms are removed, it is then time to direct special treatment to it; and this will always be much more certain and easy, when the ground, so to speak, is well prepared.

*The inflammation induced by an incision of the tissues over any swelling is often followed by a resolution of the morbid state.*

I have long since proved to the surgeon that he may with perfect safety make flaps with lardaceous tissues, provided they are neither scirrhous nor softened, and that the inflammation, which attacks them, is often sufficient to bring them into a normal condition—sometimes in the course of a few days. I had read in the writings of *Ambrose Paré* that that great surgeon was in the habit of treating the callosities of ulcers by scarifying them freely: and it occurred to me that this excellent precept, hitherto far too much neglected, was capable of considerable extension.

I will tell you a case which occurred to me some years ago. I had nearly dissected out a tumor, when the patient resolutely refused to permit the operation to be completed: I was therefore obliged to leave the tumor in its place, and lay the flaps of the wound down. In a few days the man left Paris, and I lost sight of him. Six months afterwards, he met me; there was no longer any tumor visible, although nothing had been done, since he left the hospital.

You know that ordinary hydro-sarcocele is as readily cured by puncture and injection as simple hydrocele. A patient was admitted with a transparent hydrocele, which was punctured; but no fluid escaped. I therefore incised the tumor: the contents of the sac were of a gelatinous consistence, and were contained in numerous small cells; the testicle was much enlarged and extremely hard, but smooth:—should it be removed?—I thought not. In the evening I visited my patient; a very violent inflammation had already set in; forty leeches were therefore applied above the wound, and when they ceased bleeding, poultices were applied. Next day, the inflammation was much abated; thirty more leeches were ordered. In the course of a day or two, the swelling of the testicle began to diminish; the subsidence gradually continued for several weeks, until at length the organ resumed its healthy size: the wound healed, and the patient was cured.

*Encysted serous tumor in the neck; extirpation after being laid open by incision.*

The tumor extended from the mastoid process four inches down the neck, and was situated over the course of the large blood-vessels. I first opened the cyst along its entire length, and then proceeded to dissect it from its adhesions: it was thick, hard, and very resisting. Now when the parietes of an encysted tumor are so,—a circumstance which is readily ascertained when it is fairly exposed—I advise you, contrary indeed to the instructions in surgical books, to lay it freely open, before extirpating it. You will find this easier than if you attempt to dissect it out entire. A well known fact may teach you the truth of this:—cannot you dissect the peritoneum from off the abdominal muscles with greater ease when the abdominal cavity is laid open, than when it is entire? As I proceeded in the extirpation of the cyst, I found that it very firmly adhered posteriorly to the large blood-vessels of the neck. What was to be done? If left in the wound, the probability was that a troublesome fistula might supervene. I therefore ordered my assistant to seize hold of it firmly and draw it out, while I divided fibre by fibre with the greatest caution. Success followed the attempt; it was extracted entire; and ultimately the wound was healed in the course of a fortnight.—*Bulletin de Therapeutique.*

---

### ON THE HISTORY OF THE NEW OPERATION FOR THE CURE OF SQUINTING.

Although it is admitted by all, that Professor *Dieffenbach* of Berlin has unquestionably the merit of having first actually performed the operation of dividing the internal rectus muscle of the eye for the cure of squinting, it would seem that several surgeons had previously suggested and even recommended it. The following observations are communicated by M. *Carron du Villards* in a recent Number of the Bulletin de Therapeutique.

With a natural partiality for *soi-meme,* which we suppose is common to all authors, he commences by alluding to a memoir, entitled " Practical Considerations on Sanguineous Effusions into the Eye and its Appendages," and published by him in the Gazette Medicale for 1838. In it he has reported the case of a gentleman, who, in consequence of a gun-shot wound of the eye by which one of its oblique muscles was divided, was cured of a squint which had existed for many years.

" Here," says he, " as in the case of the operation for excision of the lower jaw, a gun-shot wound had opened a new path to operative surgery. Without doubt if, at this period, I had made public some facts which I then communicated to M. *Scouttetten,* head surgeon of the military hospital at Metz, I should have reconciled the inventors of the new operation (j'aurais mis les inventeurs d'accord)."

In the Annales d'Oculiste for June (1838, we suppose) we meet with the following sentence: " It is now some time since an Italian physician suggested that squinting, attributable to the spasmodic contraction of one of the straight muscles of the eye-ball, might probably be cured by the division of the contracted muscle." M. *Cunier,* the Editor of the Annales, received this suggestion in 1837, at Montpelier from Dr. *Baschiere,* who wished him at that time to perform the operation on a relative of his own: but here the affair dropped.

M. *Jules Guerin* also, in the same year also, 1838, seems to have performed several experiments in the presence of M. *Seutin* and others: " he fell however, adds M. *Carron,* " into an error very common among scientific men; to wit, that of *thinking aloud,* before having taken the necessary steps to secure his property to himself." (Is this remark intended as a reproach against M. *Seutin* for malhonneteté ?)

In 1838, M. *Stromeyer* of Hanover, published the following remarks in his work on the cure of deformities.

" Various trials on the dead body lead me to recommend the following operation for the cure of squinting. Let the sound eye be closed, and the affected one be directed as much as possible outwards. Lay hold of the conjunctiva with forceps, and divide it at the inner canthus with a cataract knife. If the eye be drawn sufficiently outwards, the tendon of the internal rectus will be seen; a probe may then be passed under it, and with a pair of curved scissors it may be divided across without difficulty. The sound eye should be kept closed for some time, to enable its fellow, on which the operation has been performed, to recover its normal movements.

Orthopædic surgery shews that it is sufficient to divide a muscle to remove any spasmodic contraction with which it may be affected, and to enable it to resume its healthy functions; and, as to the operation which we recommend, it cannot be attended with more danger than follows the extirpation of an encysted tumor of the eye-ball."

When *Dieffenbach* first made known the results of his operation, M. *Guerin,* to whom we have already alluded, addressed to the Academy of Medecine at Paris a letter, in which he stated that for a length of time he had publicly professed his conviction that squinting was owing to a retraction of one or more of the muscles of the eye, and had distinctly announced his intentions of extending the same principle of treatment which he had for many years applied for the removal of deformities in other parts of the body. So convinced was he of the justness of his views that, to one of the most distinguished members of the academy, he had said that *squinting is the club-foot of the eye.* M. *Guerin* admits that one of the causes which had induced him to defer putting his designs into practice, was the fear of inflammation supervening in an organ, so near to the brain as the eye is.

As an historical document, the following extract from a letter published in the *Annales d'Oculiste* deserves to be given........ " Four years ago, M. *Gensoul*, ex-surgeon in chief of the Hôtel Dieu at Lyons, suggested the practice of ocular myotomy in a case of squinting; this point in surgery occupied his attention for a long time. The proposal was no secret; it was known to all the pupils of the hospital. I have myself seen M. *Gensoul* perform the operation several times on the dead body, and have heard him frequently allude to the subject. In the course of the year 1838, M. *Gensoul* visited Germany, and conversed with the surgeons of Berlin, and with M. *Dieffenbach* among the rest, on the subject, of myotomy for the cure of squinting."

But passing from the history of the operation to that of its success, it would seem that it has been much more fortunate in some countries than in others. *Dieffenbach* tells us that he has succeeded in every case; and M. *Van Roosbroeck* of ———, in Belgium, boasts of having operated on 180 persons with complete success. In France, we must confess that we have been much less fortunate; we have given a fair trial to it, says M. *Carron du Villards;* but hitherto no surgeon of good faith will pronounce the operation to be " un moyen curatif constant." M. *Roux* has failed in every case that he has tried it; M. *Velpeau* has succeeded only in two out of seven cases; M. *Guerin* himself in one out of four; and of five cases in which I (M. *Carron*) have operated, one only proved quite successful, another partially so, and in the other three no benefit whatever was obtained.

The dexterity of our surgeons will not be questioned by any one; and we should therefore very much like to be furnished with a key to the extraordinary success of M. *Van Roosbroeck*, and some other foreign surgeons. It has always appeared to us to be a heresy in surgery to apply a single and exclusive practice to the treatment of a disease of which there are numerous and different forms. Certainly, if squinting was invariably owing to the spasmodic contraction of one or more of the muscles of the eye, the division of the affected muscle or muscles might be followed with success in the majority of cases. But unfortunately such is not so, and the deformity is often attributable to conditions in which such an operation is utterly inapplicable. The opinion of M. *Venier* seems to us to be quite correct—that the only form of squinting, which we can reasonably hope to remove by the operation, is that which is permanent, and is caused by the excess of action or the deficiency in the length of the muscle, in the direction of which the deviation is present.

But whether this be correct or not, every one will surely admit that we must have more time and a larger experience before we can solve many questions relative to the operative treatment of squinting—questions which have been entirely overlooked in the recent competition among surgeons who should be the first to operate. M. *Carron* closes his remarks by alluding to a very interesting case which occurred some years ago in the practice of M. *Lisfranc*. This celebrated operator found it necessary to dissect deeply into the orbit at the inner canthus for the purpose of extirpating a cancerous tumor imbedded there; the rectus muscle was divided, and the effect of this was that the eye-ball was immediately drawn outwards. When, however, the wound was healed, the divided muscle seems to have re-united; for the eye recovered its normal axis.—*Bulletin de Therapeutique for August* 1840.

*Remarks.*—Some of the preceding observations are well deserving the attention of medical men: they may possibly check the somewhat vehement readiness, which has prevailed in this country during the last twelve months, to operate in every case of squinting indiscriminately: it has been quite a *furore*. We should like now to see a faithful table of the results of a considerable number of the operations performed six months ago. In how many of the cases has the cure of the deformity been permanent?—*Rev.*

---

### Observations on Squinting, and the New Operation for its Relief.

We find, as might have been expected, a number of communications on this, at present, popular subject in the French Journals; but certainly there does not appear to be the same unanimity as to the advantages of the new operation for the relief of the infirmity among their writers, as prevails among the numerous correspondents on the subject in the English periodicals. We rather fear that our countrymen have been somewhat premature in their announcements of the almost uniform success which has attended the division of the internal rectus muscles in the many hundred cases which have already been reported. Have the cures been really permanent? That is the important question; for it may readily be supposed that the eye may recover at the moment, and for some time after the operation, a straight direction; but that the former obliquity may return when the cicatrisation has been completely effected. This question has been examined with considerable ability by M. *Sedillot,* in a paper published in the 109th number of the Gazette des Hôpitaux.

.........."Under the general term of squinting," says he, "there are various forms of deviations of the eyeball described. Thus the eye may be drawn inwards (strabismus convergens,) or outwards, (divergens,) or upwards, (sursum vergens,) or the axis of one eye may be directed upwards and that of the other downwards, (s. horrendus.)

All these varieties of squinting may be either permanent or momentary, and also either congenital or acquired. The same eye has been observed to squint in one direction for some time, and subsequently to squint in the opposite direction. In the majority of cases, the affected eye resumes its rectitude whenever the sound one is closed; but occasionally this is not the case, and the squint remains permanent under all circumstances. The sight of the distorted eye is usually more weak than that of its fellow. Now it is important to keep in mind these several circumstances in considering the new operation which has attracted so much attention of late for the cure of this infirmity."

It was the opinion of *Buffon* that squinting was the effect of an inequality in the visual powers of the two eyes, and that any attempts to remedy the evil must be based on the re-establishment of a perfect equality.

The person, he said, to avoid seeing objects double—which will necessarily be the case if he looks at them with both eyes, the axes of which diverge—gets into the habit of looking at them steadfastly with one eye only, and tries to turn the other one away. If this be persisted in, a confirmed squint will probably be the result. During the progress of amaurosis some patients begin to squint, in consequence of the confusion of the sight arising from the unequal powers of the two eyes.

"But we must not attach too much importance," says M. *Sedillot,* "to these observations; for nothing is more common than to meet with persons, whose eyes are so different that they require one of the glasses of their spectacles to be convex and the other to be concave; and yet these persons do not squint when they lay their spectacles aside. Again, how often does it happen that a person becomes almost completely amaurotic in one eye, without having been at all aware of any gradual decay of its vision. We may, therefore, fairly conclude, that squinting is not owing solely to an inequality in the visual powers of the two eyes, but depends, in a great number of cases at least, on some other cause. May we not suppose that the feebleness of the distorted eye is referrible to a defect of action, and that, as soon as the organ is no longer kept withdrawn from the visual rays, it will gradually recover its normal functions?"

M. *Sedillot* proceeds to offer some comments on the operation of dividing one or more of the muscles of the eyeball for the relief of the infirmity in question.

It has, with considerable shew of reasoning, been argued that, unless the muscles be permanently contracted, so that the squint continues when the sound eye is closed, no good can be expected from their mere division. What advantage, it has been said, can result from lengthening a muscle which has already its normal length?

But we must not, says M. *Sedillot*, allow ourselves to be too much influenced by this objection, although we admit that it has considerable force. It requires but the least degree of predominance of action in one muscle over another to draw the organ to one side; and, although the contraction be altogether of a nervous or spasmodic nature, and be not at all connected with any structural change of the contracted muscle, yet if continued for a length of time, the external muscle, by being kept in a state of forced extension, will gradually lose its energy, and becomes at length unable to resist, by any voluntary action, its antagonist and the obstacles to its movements, which will be established in the surrounding parts.

"Were we," says M. *Sedillot*, "to name the cases in which theory points out the best chances of success, we should certainly regard the permanent squint, unaccompanied however with any inequality in the vision, as the most favourable condition for performing the operation; than the ordinary squint, in which the affected eye recovers its straightness when its fellow is closed; and last of all, that form in which the visual powers of the affected eye are more or less altered."

There cannot be a doubt but that many of the alleged cures from the performance of the operation have been prematurely reported; in not a few the squinting has returned within a longer or shorter time afterwards. M. *Guerin* himself, so zealous an advocate of the operation, acknowledges that the section of the internal rectus muscle alone is often not sufficient for the removal of the deformity, and that it is necessary to divide, at the same time, the oblique muscles, which are, it is known, in part adductors of the eyeball, and which, therefore, will sometimes keep it drawn inwards, even after the internal rectus has been divided.

It is certainly rather surprising that *Dieffenbach*, of Berlin, the first who performed the operation, and also M. *Phillips*, of Liege, both of whom have operated in a vast number of cases with an almost uniformly perfect success, according to their own reports, have not made any allusion to the necessity of ever dividing the oblique muscles. Other surgeons have been far less fortunate; indeed, MM. *Roux* and *Velpeau*, high authorities in operative surgery, seem to have met with so little success in their practice, that they do not hesitate to question the accuracy of many of the reports which have been given to the public. I myself, says M. *Sedillot*, know of a considerable number of cases, in which the operation has failed of curing the squint, and I have heard M. *Guerin* state, in his clinical conferences, that the section of the internal rectus muscle alone is not, in every case, sufficient to rectify the deformity, and that it is then necessary to divide one or both of the oblique muscles, which are, as already stated, in part adductors of the eyeball. If such be the case, the operation is far from being always so simple as many of its advocates have represented; and we cannot but feel surprised that *Dieffenbach* and *Phillips* should not have alluded to this circumstance.

Perhaps the following remarks, from the second memoir communicated by the former of these surgeons to the Royal Academy of Medicine, may be acceptable to the reader.

1. In persons who squint with one eye only, and this inwardly, the pupil is very often observed to be dilated, while that of the sound eye is considerably contracted. When this is the case, vision with the two eyes is double; and sometimes it is also double in the distorted eye itself.

2. When the internal rectus muscle is divided, the pupil becomes contracted; and should the contraction be equal to that of the pupil of the other eye, the vision is regular. But, on the contrary, if the two pupils are unequally contracted, the vision remains or it becomes double; this state being observed for the first two or three weeks after the operation, and afterwards gradually ceasing.

3. The tendon of the superior oblique muscle having been divided in several cases, where the eye was drawn upwards and inwards, the eyeball was observed to fall and become placed in the middle of the opening of the eyelids.

4. When the squint is divergent or outwards, the section of the external rectus muscle is sufficient usually to remove the deformity; but it not unfrequently happens that the eyeball becomes drawn inwards, and then it is necessary to divide the internal muscle also before its straight direction is completely restored.

5. In cases of squinting upwards, the superior rectus muscle has been divided; but this operation is of much more difficult execution.—*Gazette des Hôpitaux.*

---

### Considerations on the Radical Cure of Herniæ.

For several years past, the attention of surgeons has been directed to discover a safe and effectual method of obtaining a radical cure of reducible herniæ. It is almost unnecessary to state that all the proposals to effect this most desirable object by the mere external application of astringents and such like remedies have proved on trial quite nugatory, and that one or two of the operations which have been suggested are not without considerable danger. Castration has been more than once performed by educated surgeons as well as by several noted charlatans; but surely, apart from the circumstance of this operation not always proving successful, few patients will submit to such a mutilation. The cauterisation of the coverings of the sac, either with the hot iron or with strong chemical escharotics, has been repeatedly tried; but there are very strong objections to this method; for not only has it caused death in some instances, and the loss of the testicle in others, but it has also completely failed in other instances in preventing the subsequent descent of the hernia. The same remarks are applicable to the proposals, which have been made at different times, to use ligatures or sutures in various ways to the tumor. Then the method by incision, as in the operation for strangulated hernia, found several advocates, when M. *Petit* had the misfortune to find that it is sometimes followed with fatal consequences, independently of its being ineffectual. By associating with the incision the dissection and excision of the sac, the operation was rendered considerably more dangerous and not much more sure. There were certainly greater chances of success by the plan proposed by some surgeons, to fix in the inguinal canal, in the form of a plug, either a portion of the sac previously dissected, or a portion of the omentum, or the testicle itself; but, besides that the dissection of the sac is a tedious and painful operation, and one not altogether free of danger; besides that the testicle brought up to the ring is not easily secured in its place there and might become the seat of intense suffering; besides that the omentum, if artificially fixed, would certainly in most cases give rise to severe pains and probably enteritic disease, we know that even after such operations we cannot count upon a radical cure of the hernia. Still prosecuting their researches, other surgeons proposed to cauterise or scarify the inner surface of the sac in the canal; but, as for this purpose all the envelopes of the sac must be previously exposed and divided, it is scarcely necessary to say that such proceedings must be accompanied with no inconsiderable danger.

Things were in this state twelve or fifteen years ago, when, in spite of the anathemas of *Sabatier*, *Dessault* and *Boyer* against all attempts to effect a radical cure of hernia by means of operation, several surgeons again began to make some experiments on this subject. Permanent compression, kept up for several months successively, was again fairly tried, and several cases were published in which a cure seemed to be, and probably was, effected by the adoption of this means along with the local application of astringent washes and confinement to the horizontal posture. But this plan is too tedious and troublesome ever to be generally used.

A distinguished surgeon of Paris, M. *Belmas*, devised a plan to obliterate the herniary sac without leaving any wound in the integuments. He proposed to introduce, by means of a canula in form of a trocar, a small portion of gold-beater's skin into the serous tunic of the hernia, which was to be left there so as to excite an exsudation of plastic lymph, and thus cause the *trajet* of the hernia to be firmly plugged up. Although a few cures have been effected by this mode of procedure, M. *Belmas* has more recently substituted for the gold-beater's skin, which he used in his first experiments, minute filaments of animal matter, which he inserts and deposits in the root of the herniary sac by means of a small larding-pin (lardoire).

Mr. *Jameson*, an American surgeon, has reported a case in which he effected a cure in the following manner. The herniary envelopes being divided, as in the operation for strangulated bowel, he cut a portion of the integuments in the form of the blade of a lancet, and folded it back upon itself, securing it in the ring so as to plug up its *trajet*. We are not aware that this plan has ever been tried in France; it may indeed succeed, but it cannot be altogether free from danger.

M. *Gerdy* has proposed another method, which is certainly more safe, but is unfortunately much more uncertain. It consists in pushing up along the inguinal canal a fold of the integuments of the scrotum, in the form of the finger of a glove, and then securing this fold in its new situation by transfixing it with a strong thread by means of a needle contrived for the purpose. This operation has now been performed a good many times; but not only has it failed in several instances, but it has even been followed by fatal consequences in more than one case.

As to the proposal of M. *Bonnet* of Lyons to transfix the sac with several needles to be left in for some days, and that of M. *Mayor* to substitute strong threads, like a seton, in place of the needles, M. *Velpeau* is of opinion, that in neither of these ways can we hope to effect more than a partial closure of the sac, and therefore that we cannot count upon a radical cure of the hernia.

In 1836, M. *Velpeau* tried the effect of iodine injections into the sac of a reducible hernia, and repeated the experiment on other two patients; but the dificulty of reaching the sac with certainty, and the unsatisfactory results obtained in these three cases, are serious objections to the practice; and M. *Velpeau* himself, in the last edition (1839) of his work, alludes to it without approbation.

During the course of last year, he tried another method in two cases. This consisted of three acts or elements: a subcutaneous *incision* on the principles so ably insisted upon by M. *Guerin* in the surgical treatment of so many diseases, *scarification* of the interior of the sac and especially of its internal aperture, and lastly, *compression* of the entire length of the inguinal canal. The left fore-finger being first pushed into the external inguinal aperture to the depth of half an inch or so, a bistoury is slided along the nail and inserted through the integuments in a direction backwards and outwards; the finger is then withdrawn and the cutting edge of the instrument turned against the iliac parietes of the abdomen, and in such a manner as to make numerous scarifications in various directions, without endangering the epigastric artery: the bistoury is then withdrawn by the small entrance-wound, and the operation is completed.

In two cases M. *Velpeau* has succeeded in effecting what he has reason to believe will prove a radical cure of the hernia by adopting this practice; but further experience alone can determine whether it will be generally useful.—*Bulletin de Therapeutique.*

---

### A New Method of inducing Artificial Labour.

Dr. *Meissner* of Leipsic, whose practice has been immense for the last 20 years, objects to the plans which have hitherto been in use for the purpose of inducing premature labour, on the grounds of their insufficiency in some cases, and of their troublesome and even dangerous effects in others. The puncture of the membranes, by giving a premature discharge to the waters, is often followed by the death of the child; the internal use of the ergot of rye in large doses also exerts a hurtful influence on the life of the fœtus; while the insertion of pieces of sponge, gradually enlarged in size, into the cervix of the uterus, is at best a very tedious process, and not unfrequently causes a troublesome irritation of the uterus.

The plan, which M. *Meissner* has adopted with uniform success, as well for the infant as for the mother, is the following: he punctures the membranes high up above the cervix uteri. For this purpose he uses a long curved canula which is provided with a blunt round-pointed trocar; this is cautiously introduced, the convexity turned backwards or to the concavity of the sacrum, along the forefinger of the left hand,—the patient standing and the accoucheur kneeling before her—up to the posterior part of the cervix; it is then to be passed slowly and gradually into the uterus between its parietes and the membranes of the ovum, without separating these too much from their loose attachment. When the extremity of the canula has reached to about O. 271 of a *metre* (seven or eight inches?) above the cervix, the blunt-pointed canula is to be withdrawn and a sharp-pointed one is to be introduced. Before pushing it on, the operator should endeavour to ascertain by tact whether the point of the instrument is resting on a solid or on a yielding elastic body: if on the former, it will most probably be some part of the fœtus; and then the point of the canula should be gently displaced, before the trocar is made to penetrate the membranes. The trocar is then to be withdrawn from the canula, and, when about a tablespoonful or so of the liquor amnii has escaped, it may be withdrawn also. An occasional oozing continues from the vagina, and, usually in from 24 to 48 hours after the operation, the uterine contractions come on and expel the child.

The following cases are adduced.

*Case* 1.—A rachitic woman had been twice delivered of a child by means of the perforator and crotchet. On her third pregnancy, Dr. *Meissner* resolved to induce premature labour,* according to the plan which we have now described. This was done at ten o'clock one morning, and next day at four in the afternoon labour-pains set in, and the child was delivered by nine in the evening. In consequence of the mother not being able to suckle the infant, it died a few weeks afterwards.

---

* The author never induces artificial labour before the thirty-sixth week of pregnancy.

(This is the only case in Dr. *Meissner's* practice where the child has died; he very properly remarks that children born before the full period invariably require to be suckled. To bring them up in any other way always fails.)

*Case* 2.—A lady, 29 years of age, and decidedly rickety, had been on three successive occasions delivered by means of embryotomy. In the 36th week of her fourth pregnancy, Dr. M. perforated the membranes high up, as recommended above: a large quantity of the waters flowed out. Four and thirty hours after the operation, the pains of labour came on; when the os uteri was well dilated, the coccyx was found to be presenting. After the lapse of another hour, the feet were extracted; the head being detained at the inlet of the pelvis, it was necessary to apply the forceps. The child was however born alive, and, being suckled by the mother, became a healthy infant.

*Case* 3.—The same woman became again pregnant on the following year. On the 15th November, in the 36th week of gestation, Dr. *Meissner* punctured the membranes very high up; during the night a quantity of water escaped; but labour-pains did not come on till fifty-eight hours after the operation. These continued for five hours; and then the child was delivered without artificial assistance. It at once took to the breast, and subsequently thrived quite well.*

In the following case, the operation was performed under peculiar circmstances; it was not because there was any contraction of the usual size of the pelvis, but in consequence of the mother labouring under a severe attack of an acute disease—a new indication for inducing premature delivery by artificial means.

*Case* 4.—A woman, who had borne several living children, was again pregnant in 1838. When near the end of the eighth month of gestation, on the 23rd of May, she fell down stairs, and received a severe wound on the sacrum. A violent attack of inflammation of the kidneys ensued. She was bled from the arm, and a number of leeches applied to the loins, and took frequently-repeated doses of calomel.

On the 26th there was still great tenderness of the abdomen, accompanied with distress of breathing, and vomiting of whatever she swallowed: she was again bled.

Next day, the symptoms continued unrelieved. Dr. *Meissner*, unwilling to push the use of antiphlogistic remedies further in consequence of the pregnancy, determined to induce delivery, as he hoped that, by the distention being removed, and by the subsequent discharge of blood from the uterus, the abdominal symptoms might be greatly relieved. On the 28th, therefore, he punctured the membranes in his usual manner: in five or six hours afterwards labour-pains commenced, and rapidly became more and more violent until

---

* This woman again became pregnant, and Dr. M. once more induced premature labour. This time he experienced considerable difficulty, in consequence of not being able to detect with the point of the canula any elastic and yielding point of the membranes, where he could push in the trocar. At length he felt what he considered to be a safe point and made a puncture. No water, however, flowed out at first; but during the subsequent night a small quantity escaped. Labour came on eighteen hours afterwards; but, in consequence of the head being long detained, the forceps was applied, and delivery was safely effected, without any danger to either mother or child. The difficulty in the present instance was, in all probability, owing to the small quantity of the liquor amnii that existed.

delivery of a healthy child was effected. The patient soon found herself much relieved in every respect, except in the distress of breathing. A few doses, however, of opium and ipecacuan speedily set her all comfortable. She rapidly recovered, and was able to suckle the child: both ultimately did quite well.

Several other cases are reported in Dr. *Meissner's* memoir; but it is unnecessary to particularise any but one, in which the woman, in consequence of great deformity of the pelvis, had been already four times delivered by means of embryotomy. In her fifth pregnancy, Dr. M. resolved to induce artificial labour in the 36th week of parturition. He experienced more than usual difficulty in introducing the canula in consequence of the deformed shape of the abdomen and pelvis; the os uteri being not easily discovered per vaginam. At length it was passed up between the walls of the uterus and the membranes; but it was not possible to puncture these (the membranes) in the usual place, posteriorly, in consequence of there not being sufficient space near the vulva to press the handle of the instrument far enough back towards the sacrum. The operator was therefore obliged to seek for a point on the left side: he succeeded after a good deal of difficulty. Fourteen hours afterwards, pains commenced; but as the labour proved very tedious, the forceps was applied, and a living child was extracted.

The preceding facts cannot fail to attract the notice of all obstetricians; and we shall therefore wait to learn the results of the practice, now recommended, in the hands of different physicians. The success of Dr. *Meissner* is a sufficient evidence of the safety of the operation, when skilfully and cautiously performed. It is based on sound physiological principles: and, if once fairly established in practice, will redound much to the credit of the inventor. We may again repeat, that Dr. *Meissner* never performs it till the 36th week of gestation; that he recommends that the woman should be standing up during the operation; that he does not allow more than a spoonful or so to flow out from the canula; and that he does not employ any other means to induce the contractions of the uterus.

We are informed that another physician of Leipsic, Dr. *Guntz,* has already performed the operation several times, and met with the same success that has attended the practice of Dr. *Meissner.—Medicinische Annalen, et Gazette Medicale. Fevrier,* 1841.

---

### Results of Re-vaccination in the Deaf and Dumb Institution of Paris.

The number re-vaccinated was 128—124 pupils, whose ages varied from ten to eighteen years, and four adults, servants of the institution. Of the entire number, 60 were males and 68 were females. The operation was performed from arm to arm; the vaccine lymph was abundant; and the number of punctures made in each arm varied from two to six.

In 25 of the individuals, there neither were any traces of previous vaccination on their arms, although they had no doubt been vaccinated in infancy, nor were there any marks of small-pox on their face or body. (The mere absence, however, of cicatrices cannot be taken as a proof that the parties had never been vaccinated, nor had passed through variola.) Of these 25 cases, the vaccination produced no vesicles in 18; imperfect or false vesicles in four; and genuine cow-pox vesicles in three only.

Of seven individuals, who had distinct marks of small-pox on their faces, limbs and bodies, the operation succeeded perfectly in two, and failed altogether in five of them.

In the remaining 94 cases, there were distinct cicatrices of a former vaccination, the number of these varying from one to four or six; in some, one or more cicatrices were observed on each arm, in others on one arm only.

Now of these 94 persons, ten exhibited distinct cow-pox vesicles (after the re-vaccination,) 16 imperfect or bastard vesicles, and, in the remaining 70, the operation failed in producing any effects.

If we take, therefore, the entire number of persons "all well re-vaccinated by me," says M. *Meniere*, the reporter, "we find that in 15 cases only out of the 128, regular cow-pox vesicles were formed over the punctures on the arms; in 20 the vesicles were imperfect or bastard; and in 93 none at all were developed. From these data it appears that the operation took effect in about *one-eighth* of the whole; in about the same proportion, *one-eighth*, in those who had never had small-pox, and who exhibited no traces of vaccine cicatrices on their arms, although they had been vaccinated at some former period of life; in *one-third* of those who had had small-pox in their youth; and in about *one-tenth* of those in whom the cicatrices of a former vaccination were still distinct."

In estimating these results, it may be proper to attend to certain circumstances connected with the cases.

Of the fifteen persons in whom the re-vaccination took complete effect, ten were under thirteen years of age, and the other five were a few years older. In the two young girls, in whom it succeeded after previous small-pox, (which had left numerous and most distinct traces on the face and elsewhere) five years had elapsed in one case, and seven in the other, since the date of the attack. Among the pupils who had been vaccinated in their infancy, and in whom the re-vaccination took complete effect, two were twelve years, and the third was fourteen years old.

From these data, we may infer that the preservative or counteracting power of small-pox does not exceed that of cow-pox; since, under very similar circumstances, those who had passed through the two diseases were submitted to the same contagious influence and experienced nearly the same results.

But we are unwilling to draw any general conclusions from the preceding report; as we are well aware that experiments must be made on a much more extensive scale before we can safely do so.

In conclusion, we may state, that several infants were vaccinated for the first time from the vesicles on the arms of those in whom the second operation took effect, and that the virus thus obtained seemed to be perfectly genuine and active.—*Journal des Connoiss. Med. Chirurg. Septembre.*

---

### FRENCH OFFICIAL DOCUMENT ON RE-VACCINATION.

M. *Villeneuve*, the reporter of the Commission appointed by the Royal Academy of Medicine to examine the various reports sent from forty-one departments of France by order of the Government, has published the following Table of the general results of the vaccinations and re-vaccinations performed, and of the number and issue of the cases of small-pox in those who had been vaccinated.

| Departments. | Vaccinations. | | | Re-vaccinations after ascertained Vaccination. | | | Smallpox after ascertained Vaccination. | |
|---|---|---|---|---|---|---|---|---|
| | Number. | Successful. | Unsuccessful. | Number. | Successful. | Unsuccessful. | Number. | Deaths. |
| Aisne | 292 | 257 | 35 | 72 | 12 | 60 | 13 | |
| Alpes, (Basses) | .. | .. | .. | 9 | 2 | 7 | | |
| Ardeche | .. | .. | .. | .. | .. | .. | 1 | |
| Ardennes | .. | .. | .. | 1 | .. | 1 | | |
| Ariege | .. | .. | .. | 43 | 1 | 42 | | |
| Aveyron | .. | .. | .. | 110 | 10 | 100 | | |
| Calvados | 223 | 212 | 11 | 2 | .. | 2 | | |
| Charente | .. | .. | .. | 3 | 1 | 2 | | |
| Charente-Inferieure | .. | .. | .. | 12 | 7 | 5 | 3 | |
| Corse | .. | .. | .. | .. | .. | .. | 14 | |
| Dordogne | .. | .. | .. | 400 | .. | 400 | | |
| Eure | .. | .. | .. | 8 | 2 | 6 | | |
| Gers | .. | .. | .. | 66 | 4 | 62 | | |
| Herault | 124 | 113 | 11 | 10 | 2 | 8 | 61 | 3 |
| Ile-et-Vilaine | 1,473 | 1,392 | 81 | | | | | |
| Indre | .. | .. | .. | 361 | 2 | 359 | 1 | |
| Indre-et-Loire | .. | .. | .. | 54 | 13 | 41 | | |
| Jura | .. | .. | .. | 2 | 1 | 1 | | |
| Landes | 176 | 166 | 10 | | | | | |
| Loir-et-Cher | .. | .. | .. | 16 | 1 | 15 | 7 | |
| Loire (Haute) | .. | .. | .. | .. | .. | .. | 3 | |
| Loiret | 131 | 116 | 15 | | | | | |
| Lot-et-Garonne | .. | .. | .. | .. | .. | .. | 1 | |
| Manche | .. | .. | .. | 88 | 22 | 66 | 14 | |
| Marne (Haute) | .. | .. | .. | .. | .. | .. | 1 | |
| Meurthe | .. | .. | .. | 33 | 1 | 32 | | |
| Nord | 23,596 | 23,324 | 272 | | | | | |
| Pas-de-Calais | 53 | 51 | 2 | 41 | 6 | 35 | 20 | |
| Puy-de-Dome | 1,258 | 1,241 | 17 | 188 | 83 | 105 | 23 | |
| Pyrenées (Basses) | .. | .. | .. | 101 | 6 | 95 | | |
| Pyrenées-Orientales | 517 | 508 | 9 | 364 | .. | 364 | | |
| Saone (Haute) | .. | .. | .. | .. | .. | .. | 1 | |
| Sarthe | .. | .. | .. | .. | .. | .. | 3 | |
| Seine-Inferieure | .. | .. | .. | .. | .. | .. | 1 | 1 |
| Seine-et-Marne | 60 | 59 | 1 | .. | .. | .. | 20 | |
| Sevres (Deux) | 528 | 505 | 23 | | | | | |
| Tarn | 583 | 579 | 4 | 71 | 5 | 66 | 9 | 1 |
| Tarn-et-Garonne | 1,399 | 1,330 | 69 | 141 | 39 | 102 | 146 | |
| Var | .. | .. | .. | 2 | 2 | .. | 20 | |
| Vaucluse | .. | .. | .. | .. | .. | .. | 3 | |
| Vendée | .. | .. | .. | 1 | 1 | | | |
| Total | 30,413 | 29,853 | 560 | 2,199 | 223 | 1,976 | 365 | 8 |

*Remark.*—It is to be noticed—1, that the above table contains only the results of those reports in which the vaccinators have recorded their unsuccessful as well as their successful cases; and, 2, that wherever the re-vaccinations have been described in the reports as doubtful, they have been omitted.

It results from this table,—

1. That the proportion of cases in which vaccination failed, compared with that in which it took effect—estimated by some writers as one to eight, or one to ten—is not more than about one to fifty-four.

2. That of 2,199 cases, in which re-vaccination was performed on persons of different ages and sexes who had been successfully vaccinated at some previous period of their lives, the operation took effect in 223 cases only—which would give the proportion of about one to thirteen or fourteen.

3. That of 365 cases of confirmed small-pox, occurring in persons, who had been at some previous period successfully vaccinated, there were only eight that proved fatal—giving a proportion of about one in forty-five or forty-six.

We know that sporadic small-pox usually carries off about an eighth or a tenth of those who are affected with it; and that, when the disease becomes epidemic, the mortality is often as high as one in four, and sometimes even higher.

M. *Villeneuve,* in submitting the above table as containing the results of the labours of the Commission, admits that the data hitherto supplied are far from being sufficient to solve the question submitted by the Government to the Royal Academy—whether it is necessary to have recourse to re-vaccination as a universal measure throughout France.—*Annales d'Hygiene et de Med. Legale.*

*Remarks.*—The professional public is certainly indebted to M. *Villeneuve* for the preceding statistical table; for, although it is imperfect in several respects, it gives us the opportunity of learning the gratifying intelligence that vaccination has proved in France, as it has in every other country into which it has been introduced, one of the very greatest blessings ever bestowed by one man on his fellow-creatures. If the report told us nothing else but that small-pox after previous vaccination proved fatal in *eight* cases only out of *three hundred and sixty-five,* it would be valuable for this announcement alone.

We anxiously wait for a further and more ample account of the researches of the French Commission, appointed by the Minister of the Interior to examine the state of vaccination in France, and the various questions connected with it.

We avail ourselves of this opportunity of strongly recommending all medical men to use *fresh* and *fluid* lymph,—directly from the vesicle if possible,—whenever they practise *re*-vaccination. We are quite convinced that the failure in a large number of cases, where the operation takes no effect, is attributable to *dried* lymph having been used.—*Rev.*

---

### SKETCH OF THE PROGRESS OF MEDICINE IN FRANCE DURING 1840.

It is a useful practice, adopted by the Gazette Medicale, to recapitulate at the commencement of a new year the leading events in medical knowledge, which have been recorded during the course of the preceding one. M. *Guerin* has with this view published three somewhat lengthy papers in the January number of his journal; and although most of the notices are very brief, and quite insufficient to convey an accurate idea of the memoirs, which they profess to give an account of, yet the mere mention of many of them will often be found useful, by refreshing the memory, and by suggesting a train of thought to the mind of the reader himself. Unfortunately we have not left ourselves sufficient room to give more than an abstract of part of M. *Guerin's* first paper, and shall therefore be obliged to defer any notice of the other two papers till our next quarter.

M. *Guerin* commences with anatomy and physiology.

M. *Lallemand,* the distinguished professor of Montpellier, has endeavoured to shew that fecundation is not an act, in which an inert body is suddenly vivified by an amorphous fluid, in consequence of any electric, nervous, or dynamic influence; but that it consists essentially in the union of two living bodies, each of which is necessary to the development of the other. It is always a living part which is separated or detached from the type, either to become developed by itself, or to seek in another part the means which are necessary for its further development. The inert matter is organised and becomes living in the parent organism, before it acquires an independent existence. This idea is in accordance with the opinion of *Carus,* who maintains that the ova are formed in the ovaria of the female child even before its birth—so that, in the latter months of a pregnancy with a female fœtus, there are actually three generations co-existent in the same individual. M. *Lallemand* has communicated some interesting observations on the changes of form, which the spermatic animalculæ may exhibit in the same person, at different periods of life.......

M. *Prevost,* of Geneva, also has written upon the same subject: treating of the zoospermes, he says: "These spermatic animalcules have not the rounded extremity, as generally represented and described by most authors. They consist of—1, an anterior portion, much drawn out and very mobile, transparent, and moving with rapidity; 2, a middle portion, which is thicker; and 3, a lengthened and transparent tail, which is as moveable as the body."........

M. *Serres* has prosecuted his very interesting researches on the branchial respiration of the embryo in mammiferous animals and in birds.........

The anatomy of the nervous system has been much enriched by the labours of MM. *Bazin* and *Baillarger* and more especially of M. *Foville.* This distinguished physiologist has communicated to the Royal Academy a full account of his numerous dissections and experiments, which throw great additional light on the views, held by *Oken, Blainville,* and *Charles Bell,* on the vertebral conception or development of the head, the origin of the cerebral nerves, and the analogy between the encephalic and the spinal nervous systems. After explaining the order and mechanism observed in the development of the convolutions and the hemispheres of the brain, M. *Foville* proceeds to point out (what its vertebral conception might lead us *a priori* to infer), that the cerebral nerves are, like those of the spinal marrow, composed of two sets of filaments—the one set arising posteriorly and being exclusively sensory, and the other set arising anteriorly and being exclusively motory: the olfactory, the optic, and the auditory nerves belong altogether to the first set, arising from, or being connected with, the posterior bundles of the medulla. To demonstrate these facts, we must follow the medulla into the cranium, with the characters which it has in the vertebral canal—a task which M. *Foville* has done with the greatest success. In the concluding part of his memoir, he very ably points out and illustrates the relations which the convolutions of the brain have with the calvarium. He shews that its elevations and depresions represent outwardly the elevations and depressions of the parietes of the ventricles.

The researches of M. *Bazin* have for their object to establish the existence of certain connexions, anatomical, physiological, and zoological, between the different parts of the nervous system—to shew, for example, the relations and the manner of association of the sensory and the motory nerves in the encephalon, which the author regards as a centre where the first set terminates, and whence the second set comes out. Those of M. *Baillarger* relate almost entirely to the physical composition or structure of the brain. He has endeavoured to shew that the cortical substance alone is composed of six layers or strata, alternately white and cineritious—an arrangement which, according to his opinion, implies an analogy with the plates of a voltaic pile.*............

---

* Vide Medico-Chirurgical Review for October, 1840. Art. Memoirs of the Academy of Medicine.

M. *Longet* has, by a series of well-contrived experiments proved that M. *Majendie* has fallen into an error in supposing that the anterior or motory roots of the spinal marrow are at all connected with sensation; and he shews that the conclusions of *Charles Bell* are strictly correct.......

Dr. *M. Hall* has prosecuted his researches on the reflex functions of the nervous system with great ability, and he has been ably seconded by *Müller, Volckmann,* and *Budd*.......

M. *Fleurens* has examined with much success the mode of the growth of the bones and of the teeth. By feeding young animals on madder, he has shewn that the development of these structures takes place by the super-addition of new laminæ and the absorption of the old ones in both structures; but with this very important difference, that in the former the new laminæ are deposited on the outer surface and the inner laminæ are simultaneously absorbed; while in the latter the new laminæ are added internally, and the absorption takes place on the outer surface.......

The physiology of the circulation has not received so much attention during the last year as that of the nervous system. It may be right however, to allude to the researches of MM. *Dubois* and *Poiseuille* on the organisation of the capillary vessels and the propulsion of the blood through them; and also to those of M. *Latour,* who has shewn that the tissues of cold-blooded animals are not susceptible of genuine inflammatory action........ M. *Donné* has been prosecuting his most interesting enquiries on the intimate structure and composition of many of the animal textures, diseased as well as healthy; he is the first who has produced photogenic images of microscopic objects obtained with the ordinary microscope.

Another labourer in the field of microscopic anatomy has been M. *Gluge,* whose researches have thrown considerable light on the pathology of *ramollisement* of the brain. He has shewn that, in a great number of cases, the white softening exhibits distinct appearances of suppuration; that the grey or coloured softening, without sanguineous effusion, exhibits the products of the first degree of inflammation, the formation of compound globules; and that the coloured softening, with sanguineous effusion, may re-unite the preceding phenomena, or merely the appearance of a mechanical imbibition of a sanguinolent serum. It would seem, if M. *Gluge's* views are confirmed, that in the majority of cases the softening of the cerebral substance is owing to a certain degree of inflammation, which has never hitherto been distinctly detected. With respect to the causes of these cerebral changes, it is more than probable that in very numerous instances they are associated with, if not directly attributable to, hypertrophy of the heart.

The intimate connection between cerebral and cardiac diseases has of late years been studied with great assiduity. Every practical physician must daily meet with instances of it; although it is not unfrequently difficult to determine in head disorders whether they are actually induced, or are merely aggravated, by the co-existing disturbance of the central organ of circulation. However this may be, it is always sound practice to make ourselves acquainted with the state of the heart, whenever we have reason to suspect the existence of cephalic mischief. One of the most potent of all remedies in such cases is digitalis—especially in the form of infusion, and it is unnecessary to say that its action is especially on the heart. The infusion may be associated with minute doses of the corrosive sublimate in many cases with striking advantage.

